# Characterization of Collagen Peptides in *Elaphuri Davidiani Cornu* Aqueous Extract with Proliferative Activity on Osteoblasts Using Nano-Liquid Chromatography in Tandem with Orbitrap Mass Spectrometry

**DOI:** 10.3390/molecules22010166

**Published:** 2017-01-20

**Authors:** Yanjuan Zhai, Zhenhua Zhu, Yue Zhu, Dawei Qian, Rui Liu, Yunru Peng, Yuhua Ding, Zhen Ouyang, Jin-ao Duan

**Affiliations:** 1 Jiangsu Collaborative Innovation Center of Chinese Medicinal Resources Industrialization, and National and Local Collaborative Engineering Center of Chinese Medicinal Resources Industrialization and Formulae Innovative Medicine, Nanjing University of Chinese Medicine, Nanjing 210023, China; zhaiyanjuan1990@126.com (Y.Z.); 04040416@163.com (Z.Z.); nzyzy808@163.com (Y.Z.); dja@njucm.edu.cn (J.D.); 2 Jiangsu Provincial Academy of Chinese Medicine, Nanjing 210028, China; pengyunru@126.com; 3 Jiangsu Dafeng Milu National Nature Reserve, Dafeng 224136, China; dingyuhuamilu@126.com; 4 School of Food and Biological Engineering, Jiangsu University, Zhenjiang 212013, China; zhenouyang@ujs.edu.cn

**Keywords:** collagen peptide, *Elaphuri Davidiani Cornu*, nano-liquid chromatography-mass spectrum detection, osteoblast

## Abstract

First documented in *Shennong Bencao Jing* (about 200 B.C.–200 A.D.), *Elaphuri Davidiani Cornu* (EDC) has been recorded for its effects in strengthening bones and balancing other aspects of overall health for approximately 2000 years. In the present study, our aim was to investigate which are the components of the active EDC fraction by a peptidomic strategy. We explored the extent to which EDC increases the proliferation of osteoblasts by measuring the elevations in osteonectin and type I collagen mRNA levels and characterized it using nano-flow liquid chromatography in tandem with orbitrap mass spectrometry. In total, 272 peptide sequences from collagens were determined. “Hot regions” in parent proteins determined by peptide heat maps which indicated that amino acid sequences in the regions might undergo proteolysis easily and generate peptides. Among the identified peptides, 90.2% were hydrophilic, and the molecular weight of 97.1% of identified peptides was lower than 2000 Da. According to these results, EDC collagen-derived peptides were easily analyzed and identified. Moreover, this methodology is feasible to characterize the active peptides matrices originated from collagen hydrolysates or some other animal horn- derived TCMs.

## 1. Introduction

Animal horns are pointed projections on the head of animals, and are an important part of traditional Chinese medicines (TCMs). The antler of Milu deer (*Elaphuri Davidiani Cornu*, EDC) was first documented in *Shennong Bencao Jing* (about 200 B.C.–200 A.D.), and has reported efficacy in strengthening bones and balancing other aspects of overall health. However, most of the Milu deer in the Nanyuan Royal Hunting Garden of the Qing dynasty were killed, to be consumed by starving peasants in 1895 after the hunting garden was destroyed by a flood on the Yongding River. Then in 1900, the remaining Milu deer were shot and eaten by troops during the Boxer Rebellion, making Milu deer extinct in China [1]. Nowadays, as a species under first rank state protection in China, Milu deer are well protected. Since the reintroduction of Milu deer from the United Kingdom, the population has significantly increased from 18 to over 3000 in the last three decades in China [2]. The antlers of Milu deer are naturally shed every year, and can be easily collected, therefore, as easy obtained components, there were sufficient Milu deer antler resources for our scientific study.

Scientists are engaged in investigating the chemical components and pharmacological effects of EDC. Studies have shown that EDC contains amino acids, collagens, nucleosides, phospholipids, inorganic elements, etc. [2]. It has also been reported that EDC possesses diverse bio-activities, including anti-osteoporosis [3], anti-aging [4], and immuno-enhancing activity [5]. It is known that collagen plays an important structural function in organisms, especially in bone and cartilage. Collagen can be found in the bones, skin and connective tissue of animals. Collagen hydrolysates (CHs) are produced from collagen by breaking down the molecular bonds between peptides using chemical or enzymatic hydrolysis. CHs can improve bone metabolism and biomechanical parameters in ovariectomized mice [6]. Oral administration of CHs was demonstrated to increase the quantity of collagen and proteoglycans in the bone matrix of ovariectomized rats [7].

Like the antlers of other deer, such as the European red deer (*Cervus Elaphus*), EDC is a structure that regenerates every year. There are four different histological zones in a transversal cross section from the periphery to the center: the subvelvet zone, the zone of osteonic bone, the transition zone, and finally the central spongiosa zone [8]. The two outer regions are very dense and compact, whereas the center presents a general honeycombed structure [9]. As for other biomineralized tissues such as antler, bone, and, teeth, collagen is also the dominant protein in antler [10]. Traditional Chinese herbs are normally aqueous extracted and administered orally in clinical applications, which is typically the same for EDC. The EDC extract is a mixture of hundreds of compounds, most of which are peptides from non-specific proteolysis of collagen. It has been demonstrated that collagen peptides have an effect on bone resorption factors which supports the theory that they stimulate osteoblast activity [11]. However, a comprehensive peptide analysis of EDC extract is lacking therefore it is necessary to provide an overview of the peptide profile of the extract. Currently, proteins or peptides in a mixture can be identified using shotgun proteomics or peptidomics technology based on the generation of tandem mass spectrometry (MS/MS) [12]. In the present study, our aim is to investigate what composes the active EDC fraction by peptidomic strategy. Firstly, an active fraction of EDC (EDCF) was screened and the effects of EDCF on osteoblasts were evaluated. Subsequently, nano-flow liquid chromatography tandem orbitrap mass spectrometry (nano-LC-MS) was used to identify peptides in the fraction. We consider that this peptidomics-based approach is feasible to identify and analyze the active peptide matrix from animal-horn-derived TCMs.

## 2. Results and Discussion

### 2.1. Inducing the Differentiation Activity of EDCF in Cultured Osteoblasts

**Figure 1:**
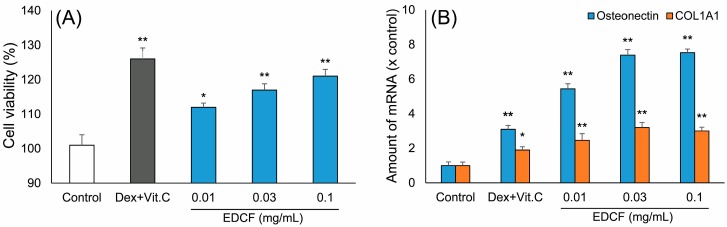
Proliferation of cultured osteoblasts induced by dexamethasone (Dex) and vitamin C (Vit. C), and by *Elaphuri Davidiani Cornu* (EDC) fraction. (**A**) Cell viability of osteoblasts; (**B**) Total mRNA levels of bone differentiation markers: type I collagen and osteonectin were quantified. Data were expressed as the fold of basal value (x basal) where the control value is set as 100% and 1 respectively, Mean ± SEM, *n* = 3. * *p* < 0.05, ** *p* < 0.01.

The osteoblasts were directly isolated from the calvarias of postnatal day 1 rats. The osteoblasts were able to undergo differentiation induced by dexamethasone and vitamin C. The methylthiazole tetrazolium (MTT) method was used to observe the effect of ECDF on the proliferation of osteoblasts. As shown in Figure 1A, the proliferation of osteoblasts was up to 112%, 117%, and 121% following incubation with EDCF at 0.01, 0.03, and 0.1 mg/mL, respectively. The EDCF-induced osteoblast differentiation was further confirmed by activation of osteonectin and type I collagen (COL1A1) mRNA levels. Using quantitative real-time polymerase chain reaction (PCR), the levels of these differentiation markers increased significantly following treatment with dexamethasone and vitamin C (*p* < 0.05; *p* < 0.01). The levels of COL1A1 and osteonectin increased by more than 2.4-fold and 5.4-fold after treatment with EDCF (*p* < 0.05; *p* < 0.01), respectively.

The process of bone development can be divided into cell proliferation, cell differentiation, cell mineralization and cell apoptosis. This progression from one differentiation stage to the next is accompanied by the activation and subsequent inactivation of transcription factors and the expression of related marker genes i.e., osteopontin, collagen type I, alkaline phosphatase, bone sialoprotein and osteocalcin in osteoblasts [13]. Osteoblasts are differentiated to improve bone proliferation. The results of the present study indicated that EDCF had efficacy not only in promoting cell viability, but also in increasing mRNA expression of vital bone differentiation markers. These findings also suggest that EDCF could have potential effects on osteoporosis.

### 2.2. Characterization of Peptides in EDCF

**Figure 2:**
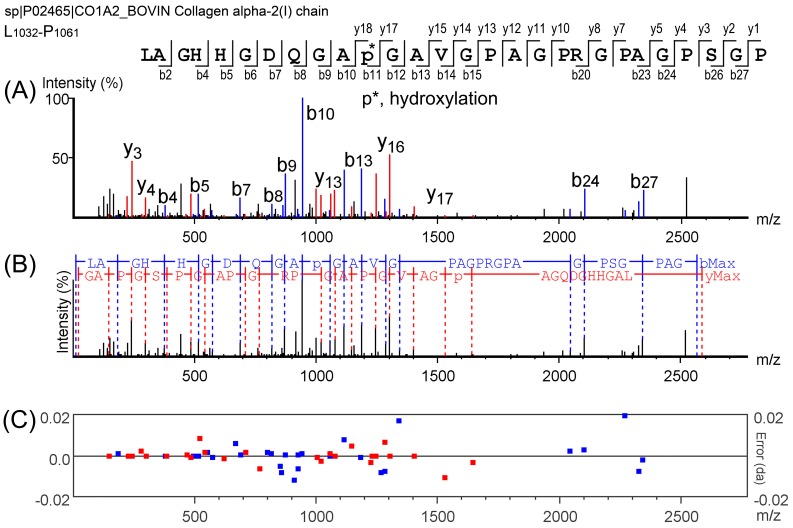
Identification of peptides based on the MS/MS spectra. (**A**) Amino acid sequence of the peptide and its MS/MS ions series, b ions and y ions were marked on the sequence of peptides; (**B**) Amino acid sequence calculated by de novo sequencing algorithm using PEAKS software; (**C**) Error of the ions. The mid line represents theoretical values of the ions, red spots represent the position of y ions, and blue spots represent the position of b ions. All errors are lower than 0.02 Da.

Nano-LC MS/MS-based peptide identification has made peptide analysis in highly complex mixtures feasible. In the present study, taking the peptide LAGHHGDQGAPGAVGPAGPRGPAGPSGPAG as an example, high energy collision dissociation (HCD) was optimized to obtain b and y ion series (Table S1, Figure 2A,B) and the error of these ions was lower than 0.02 Da (Figure 2C). These ions were then searched against the Pecora database, and the amino acid sequence was confirmed. As shown in Figure 2A, it could be confirmed that this peptide matched with collagen α-2(I) based on the database searching. As a result, a total of 272 peptides were identified in the EDCF, and the parent proteins of these peptides were collagens. It was confirmed that these peptides were hydrolyzed from these three parent proteins (unique peptides ≥ 2), collagen α-1 (W5P481) and collagen α-2 (W5NTT7 and P02465).

**Figure 3:**
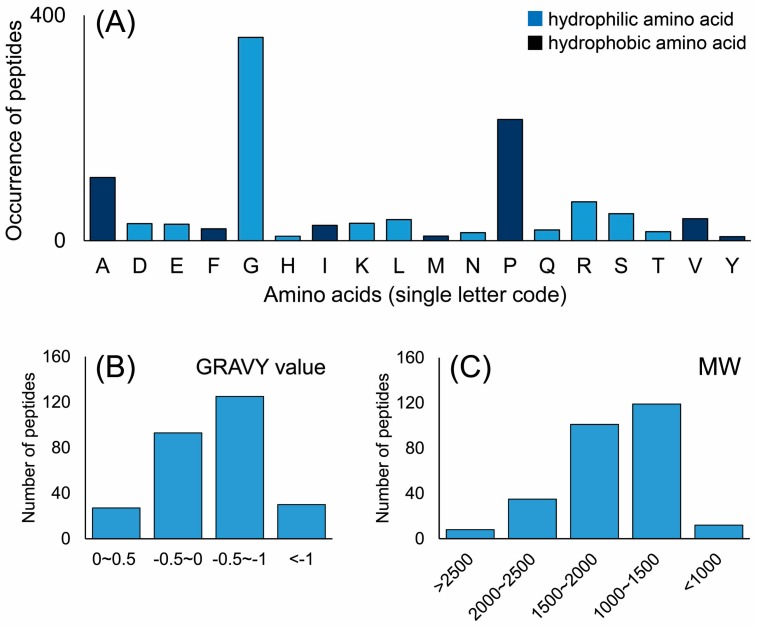
(**A**) Occurrence of the amino acids in all identified peptides; (**B**) Grand average of hydropathicity (GRAVY) index value of the identified peptides. GRAVY index values lower than 0 represent that the peptides possess hydrophilic properties; (**C**) Molecular weight (MW) distribution of the identified peptides. All of the value based on the total number of peptides.

Among these 272 peptides, the frequency of amino acid residues in each identified peptide was determined (Figure 3). The most frequent amino acid was glycine (Gly, G, 361 times), followed by proline (Pro, P, 215 times). Glycine and proline are the basic structural amino acid residues of collagens. A conspicuous Gly-X-Y repetitive sequence involves every third amino acid position occupied by a Gly and the X and Y positions are often occupied by Pro [14]. The total frequency of hydrophilic amino acids was 662, and the hydrophobic amino acids were observed 429 times (Figure 3A). Furthermore, the grand average of hydropathicity (GRAVY) index value was used to evaluate the hydrophilic and hydrophobic character of the identified peptides. As shown in Figure 3B, of the identified peptides, 90.2% were hydrophilic due to a GRAVY index value lower than 0. In general, hydrophilic amino acids frequently occurred in the identified peptides of EDCF, which may appear hydrophilic in nature.

Considering the global peptides, as shown in Figure 3C, the molecular weight (MW) of identified peptides ranged from 793.40 to 3288.66 Da, and 97.1% of the peptides were lower than 2000 Da. Active peptides usually contain 2–20 amino acid residues per molecule therefore, there may be a higher chance that the identified peptides cross the membrane barrier and exert biological effects due to their low molecular weight [15]. 

**Figure 4:**
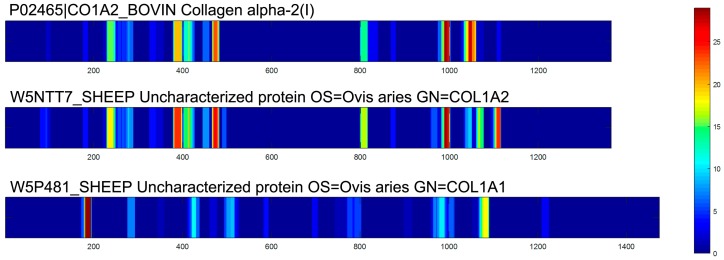
Heat maps of collagens. Heat maps graphically displaying the occurrence of the different amino acids in the primary sequence of the parent protein of the identified peptides. Blue regions indicate low occurrence; red regions indicate frequently appearing residues.

In the present study, a comprehensive analysis of the peptides was used to obtain information on the presence of peptides and the mode of peptide formation in EDCF. Primary sequences of peptides showed that most of these peptides originated from collagens, as shown in the Supplemental Data Figure S1, Figure S2 and Figure 4. MS/MS spectra of peptides were set up to search the Pecora database which contains 94,259 proteins (the Pecora are an infraorder of even-toed hoofed mammals with ruminant digestion, including deer). As a result, there are two parent proteins from which these peptides origin, collagen α-1 (W5P481) and collagen α-2 (W5NTT7 and P02465). It may be helpful to explain what happened to the EDC proteins during the aqueous extraction process. The regions which release peptides are not evenly distributed in the total protein sequences. In order to understand the locations of identified peptides, the number of times single amino acids made up the peptides in the primary sequence of proteins was counted and reported as a protein heat map. Heat maps of peptide distribution indicated the locations of highest hydrolysis of each protein, which graphically displayed the occurrence of the different amino acids in the primary sequence of the parent protein. In heat maps, as shown in Figure 4, there are particular “hot regions” in the amino acid sequence which might undergo proteolysis easily and generate peptides [16]. Blue regions indicate low occurrence, red regions indicate frequently appearing residues. In the primary sequence of proteins, each position only has one amino acid, and there were various hydrolyzed peptides which might be derived from the same protein fragment. Therefore, in the heat map, amino acids in each position would appear not only once. In contrast, there are also some amino acid sequences which do not undergo hydrolysis easily. As shown in Figure 4, according to the heat map profiles, three proteins showed different releasing types. Two types of collagen α-2(I) showed a similar profile (Figure 4). 

**Figure 5:**
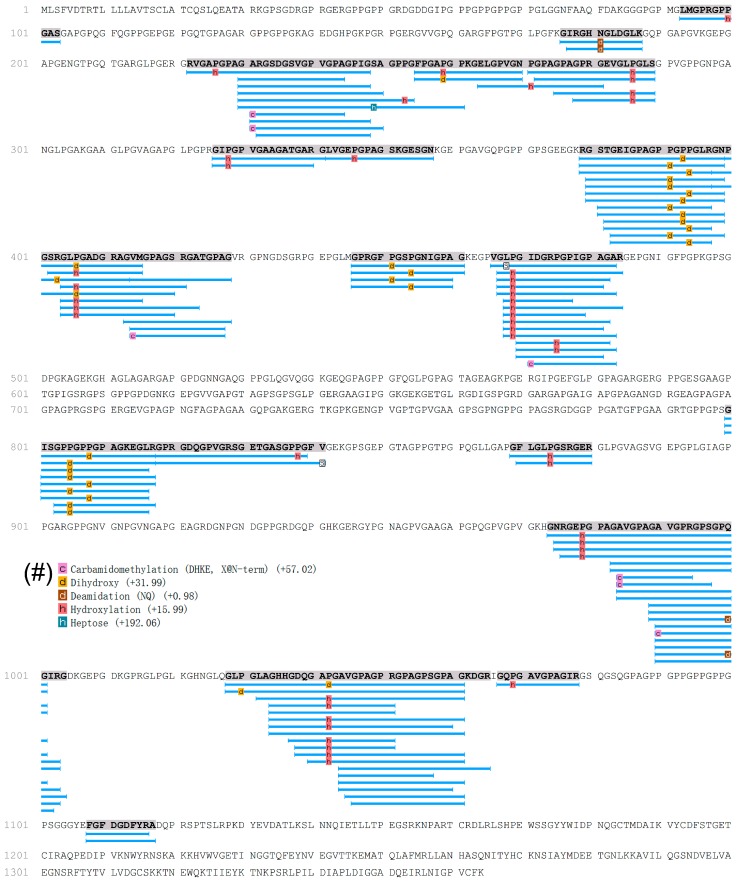
Sequence of collagen α-2(I) (P02465|CO1A2_BOVIN Collagen α-2(I) chain) and the distribution of identified peptides. (#) Post-translational modification of proteins in collagen α-2(I), “c”: carbamidomethylation; orange “d”: dihydroxy; brown “d”: deamidation; pink “h”: hydroxylation; and green “h”: heptose.

Taking collagen α-2(I) as an example to explain how these peptides are generated, the locations of 120 peptides identified from collagen α-2(I) are shown in Figure 5. Peptides were generated from seven main “hot regions” including R222 to G254, G255 to S289, R379 to G429, V466 to R484, G800 to V841, G974 to G1004, and G1028 to R1065, which were important sources of peptides. Water molecules normally occupy the space between collagens and interconnect them through hydrogen bonding [14]. As shown in Figure 5, the N-terminus and C-terminus amino acid residues of the seven “hot regions” were hydrophilic amino acids, except for V466 and V841. During the aqueous extraction process, non-specific cleavage usually occurred at hydrophilic amino acids. For example, it can be seen that the peptide fragment from R222 to G254 was first dropped from collagen and dissolved by water extraction, and a “hot region” was generated. The “hot region” was then irregularly hydrolyzed to generate peptides, such as RVGAPGPAGARGSDGSVGPVGPAGPIG, GARGSDGSVGPVGPAGPIG, and GARGSDGSVGPVGPA.

Collagen is the main structural component in bone formation. Hu et al. has pointed out that fish collagen peptide has a positive effect on osteoblasts [13]. CHs have been widely used in clinical application, and a number of studies have suggested that CHs can improve bone collagen metabolism, bone mineral density and bone mass content in rats fed a calcium-deficient diet [17]. It was demonstrated that oral administration of CHs could reduce bone loss in the bone matrix by increasing the quantity of type I collagen and increasing bone strength in ovariectomized animals [6,11,18]. CHs with molecular weight lower than 3 kDa can increase osteoblast proliferation, alkaline phosphatase activity, and COL1A1 gene expression. Similarly, the active fraction (<3 kDa) of EDC promoted osteoblast proliferation and increased the gene expression of two important markers, osteonectin and COL1A1.

The fragments of enzymatic CHs may be taken up by the extracellular matrix of chondrocytes in the processes of regeneration both in vitro and in vivo [19]. It is suspected that CHs improve bone formation and may be associated with the absorption of collagen-derived peptides thus affecting bone metabolism. After oral administration of collagen-derived peptides, some collagen derived di- or tripeptides can be detected in blood [20]. Furthermore, collagen-derived peptides stimulate osteoblast growth and differentiation, and reduce osteoclast differentiation to modulate bone formation [6].

In our previous study, there are collagen, peptides, polysaccharides, nucleosides, and nucleobases in EDC, however, the contents of polysaccharides, nucleosides, and nucleobases are very low. Compounds including 17 nucleosides and nucleobases were detected in EDC powder, and the total content was ranging from 6 μg/g to 49 μg/g [1]. The content of polysaccharides in EDCF is very low, which cannot be detected. In the present study, EDCF showed good efficacy and we speculate that other low molecular compound such as nucleosides, nucleobases or oligosaccharides might not be the active compound in EDCF, but the collagen derived peptides.

According to the traditional processing method, EDC is extracted with boiling water rather than enzymatic hydrolysis. We speculate that protein fragments or non-specific generated peptides could be obtained by non-specific proteolysis during extraction. As shown in Figure 5, hydrolyzed peptides did not show site-specific properties, and peptides were mainly generated from the seven “hot regions”. However, based on the present study, it cannot be excluded that these peptides already exist naturally in the EDC sample before extraction, and we will try to clarify it in further studies. Based on the characterization analysis of the EDCF in the present study, the peptides in EDCF were probably generated from EDC collagen fragments as chemical cleavage during extraction. Furthermore, taking up these peptides could help to improve bone metabolism both in vitro and in vivo. In addition, most peptides in the EDCF were lower than 2 kDa, which means that it might be easier to obtain EDC peptides with MWs from 793.4 to 2000 Da by a non-specific hydrolysis process. Peptides with MWs lower than 2 kDa may have more chance of crossing the biological membrane and exerting biological effects.

## 3. Materials and Methods

### 3.1. Chemicals and Materials

The samples of EDC were acquired from the *Elaphuri Davidiani* National Nature Reserve in Jiangsu Dafeng, and authenticated by Professor Jin-ao Duan from Nanjing University of Chinese Medicine. The EDC samples were then pulverized to obtain a fine powder.

Formic acid was obtained from Sigma–Aldrich Chemical Co., Ltd. (St. Louis, MO, USA). LC-grade methanol and acetonitrile were purchased from Merck KGaA (Darmstadt, Germany). Ultra-high purity water was prepared using a Millipore-Q system (Millipore Corporation, Billerica, MA, USA).

Vitamin C, dexamethasone and type I collagenase were purchased from Sigma-Aldrich Chemical Co., Ltd. MEM α basic (1×), fetal bovine serum (FBS), Tris-EDTA buffer (TE), and α-modified Eagle’s medium-α (α-MEM) were purchased from Gibco Co., Ltd. (Grand Island, NY, USA). The PrimeScript™ RT Reagent Kit with a gDNA Eraser was purchased from TaKaRa Co., Ltd. (Otsu, Japan).

### 3.2. Preparation of EDC Extract and Ultrafiltration

EDC was cut into small pieces and subsequently pulverized to a fine powder. The powder (200 g) was decocted twice with 10× quantities of water (2 L) for 6 h. The extract was filtered and the solvent was evaporated using a rotary evaporator at 60 °C; then it was lyophilized and the resulting powdered EDC extract was stored at −20 °C.

Ultrafiltration was performed using membranes with 3 kDa cut-off values on a Mini Pellicon Ultrafiltration system (Millipore Corporation). The EDC extract prepared as above was redissolved in pure water, filtered through a 0.45 μm nylon syringe filter and separated into two fractions. The fraction with a molecular weight lower than 3 kDa was collected, lyophilized, and stored at −20 °C. The lyophilized EDCF was re-dissolved in 0.9% sterile saline as needed.

### 3.3. Measurement of the Effects of EDC on Osteoblasts

#### 3.3.1. Cell Culture

The isolation and culture of osteoblasts was performed according to a previously described method [21] with some modifications. Postnatal day 1 rats were decapitated to collect calvarias. Briefly, tissues were sequentially digested with 1% trypsin for 10 min, 0.2% collagenase for 20 min, and finally freshly prepared 0.2% collagenase for a further 40 min. After digestion, the supernatant was collected and centrifuged for 5 min at 200× *g*. Osteoblasts were re-suspended and maintained in α-MEM, supplemented with 10% FBS, penicillin (100 U/mL), streptomycin (100 μg/mL), and 2 mM l-glutamine in a humidified atmosphere of 5% CO_2_ at 37 °C. The medium was changed every 3 days. Reagents for cell cultures were purchased from Invitrogen (Invitrogen Corporation, Carlsbad, CA, USA) The experiments on animals have been approved by the Animal Experimentation Ethics Committee of Nanjing University of Chinese Medicine and conformed to the guidelines of the “Principles of Laboratory Animal Care” (NIH publication No. 80-23, revised 1996). Effort was made to minimize the number and suffering of the animals.

#### 3.3.2. MTT Cell Viability Assay

Cell viability was assessed using a modified MTT assay. Briefly, osteoblasts were seeded in six-well plates at a final concentration of 1 × 10^4^ cells per well. After 24 h, the medium was removed. Differentiation was induced by treatment with vitamin C (100 μM) and dexamethasone (20 nM). The cells were rinsed with phosphate-buffered saline (PBS) and incubated with EDCF at different concentrations (10, 30, and 100 μg/mL) and dexamethasone + vitamin C (Dex + Vit. C) for 24 h. After removing the medium and washing with PBS twice, the cells were treated with MTT solution (5 mg/mL in PBS) in medium without phenol red and serum. The cells were stored in a humidified atmosphere of 5% CO_2_ at 37 °C. The liquid was then removed and dimethyl sulfoxide (150 μL) was added. Absorbance at 570 nm was read in a microplate spectrophotometer (Tecan GENios, Tecan Trading AG, Männedorf, Switzerland).

#### 3.3.3. Real Time Quantitative PCR

Differentiation was induced by treatment with vitamin C (100 μM) and dexamethasone (20 nM). After 24 h exposure to EDCF at different concentrations (10, 30, and 100 μg/mL) and Dex + Vit. C, osteoblasts were harvested and rinsed twice using ice-cold PBS. Total RNA was extracted and transcribed into cDNA using Trizol and a reverse transcription kit (TaKaRa) according to the manufacturer’s protocol. Real-time PCRs of COL1A1 and rat osteonectin were performed using equal amounts of reverse-transcribed products and carried out using SYBR Premix Ex Taq (TaKaRa). All data were normalized to the expression of the endogenous reference gene glyceraldehyde-3-phosphate dehydrogenase (*GAPDH*). The following primers were used for qPCR amplifications: COL1A1 (5′-TCCGCCGATGTCGCTATC-3′ and 5′-CAAGTTCCGGTGTGACTCGT-3′); rat osteonectin (5′-GAAGAGATGGTGGCGGAG-3′ and 5′-ACAGGCAGGGGGCAATGATTTG-3′); GAPDH (5′-GGTGAAGGTCGGTGTGAACG-3′ and 5′-CTCGCTCCTGGAAGATGGTG-3′). The real-time PCR was performed in a 20 mL reaction system by an ABI Prism 7500 sequence amplification system (AB Sciex, Concord, ON, Canada). The relative levels of transcript expression were quantified using the ΔΔCt method.

### 3.4. Peptide Characterization by Nano-LC-MS/MS

All samples were analyzed by the Dionex 3000 nano-LC system tandem LTQ-Orbitrap Velos Pro (Thermo Fisher Scientific, Waltham, MA, USA) with HCD. Lyophilized EDCF was re-dissolved in 0.1% TFA, and was desalted by SepPak C18. Desalted EDCF sample was dried by centrifugal concentration. Then EDCF sample was re-dissolved in acetonitrile/formic acid/water (2/0.2/98, *v*/*v*/*v*), and five microliters EDCF sample was loaded onto a self-packed 5 μm Reprosil C18AQ column (75 μm × 150 mm). The mobile phase consisted of acetonitrile/formic acid/water (2/0.2/98, *v*/*v*/*v*) for buffer A and acetonitrile/formic acid/water (80/0.2/20, *v*/*v*/*v*) for buffer B. Processed samples were analyzed using a 150 min gradient from 2% to 30% of B. The LTQ-Orbitrap was operated in data-dependent acquisition mode to automatically alternate between a full scan (*m*/*z* 300–2000) in the Orbitrap and HCD MS/MS scans in the linear ion trap. Helium was used as the collision gas for HCD. The normalized collision energy was 35% and the activation time was 30 ms. Unless otherwise stated, three replicate measurements were made at each MS setting. Data acquisition was controlled by Xcalibur 2.0.7 and Tune 2.4 software (Thermo Fisher Scientific).

All MS/MS spectra were extracted with Xcalibur (version 2.0.7.) and analyzed using PEAKS 7.5 (Bioinformatics Solutions Inc., Waterloo, ON, Canada) which was set up to search the Pecora_uniprot-taxonomy_35500 database (downloaded on the 28 July 2015). Spectra were also searched against an equal number of decoy sequences to estimate the false discovery rate as described previously [22]. The specified enzyme was chosen as non-enzyme and up to two missed cleavages were allowed. Oxidation of methionine (+15.9949) and acetylation of the protein N-terminus (+42.0106) were specified as variable modifications. All other parameters were default settings, including a fragment ion tolerance of 0.5 Da and a maximum precursor ion tolerance of 6 ppm after recalibration. Identification of peptides by de novo sequence was filtered to curate a dataset with a value for the Average Local Confidence (ALC) score larger than 90%.

## 4. Conclusions

In conclusion, the current study demonstrated that the fraction of EDC aqueous extract with a MW lower than 3 kDa increased the viability of osteoblasts and increased the mRNA levels of osteonectin and COL1A1. Furthermore, a workflow for the analysis of peptides in EDCF was developed, and a total of 272 peptides were identified. The non-specific hydrolysis peptides of EDCF were described and a protocol was established to investigate the complex mixture from CHs and elucidate the process of peptides formation. The present investigation elucidated complex components derived from CHs using a time-saving and labor-saving nano-LC MS/MS analysis strategy.

## Supplementary Materials

Supplementary materials can be accessed at: http://www.mdpi.com/1420-3049/22/1/166/s1.
