# Supplementary material for: Characterization of Collagen Peptides in Elaphuri Davidiani Cornu Aqueous Extract with Proliferative Activity on Osteoblasts Using Nano-Liquid Chromatography in Tandem with Orbitrap Mass Spectrometry
Source: Molecules. 2017 Jan 20;22(1):166. doi: 10.3390/molecules22010166 (PMC6155669; doi:10.3390/molecules22010166)
Supplement: Supplementary file 1 [file molecules-22-00166-s001.pdf]

Supplementary Materials: Characterization of Collagen Peptides in *Elaphuri Davidiani Cornu* Aqueous Extract with Proliferative Activity on Osteoblasts Using Nano-Liquid Chromatography in Tandem with Orbitrap Mass Spectrometry

Yanjuan Zhai, Zhenhua Zhu, Yue Zhu, Dawei Qian, Rui Liu, Yunru Peng, Yuhua Ding, Zhen Ouyang and Jin-ao Duan

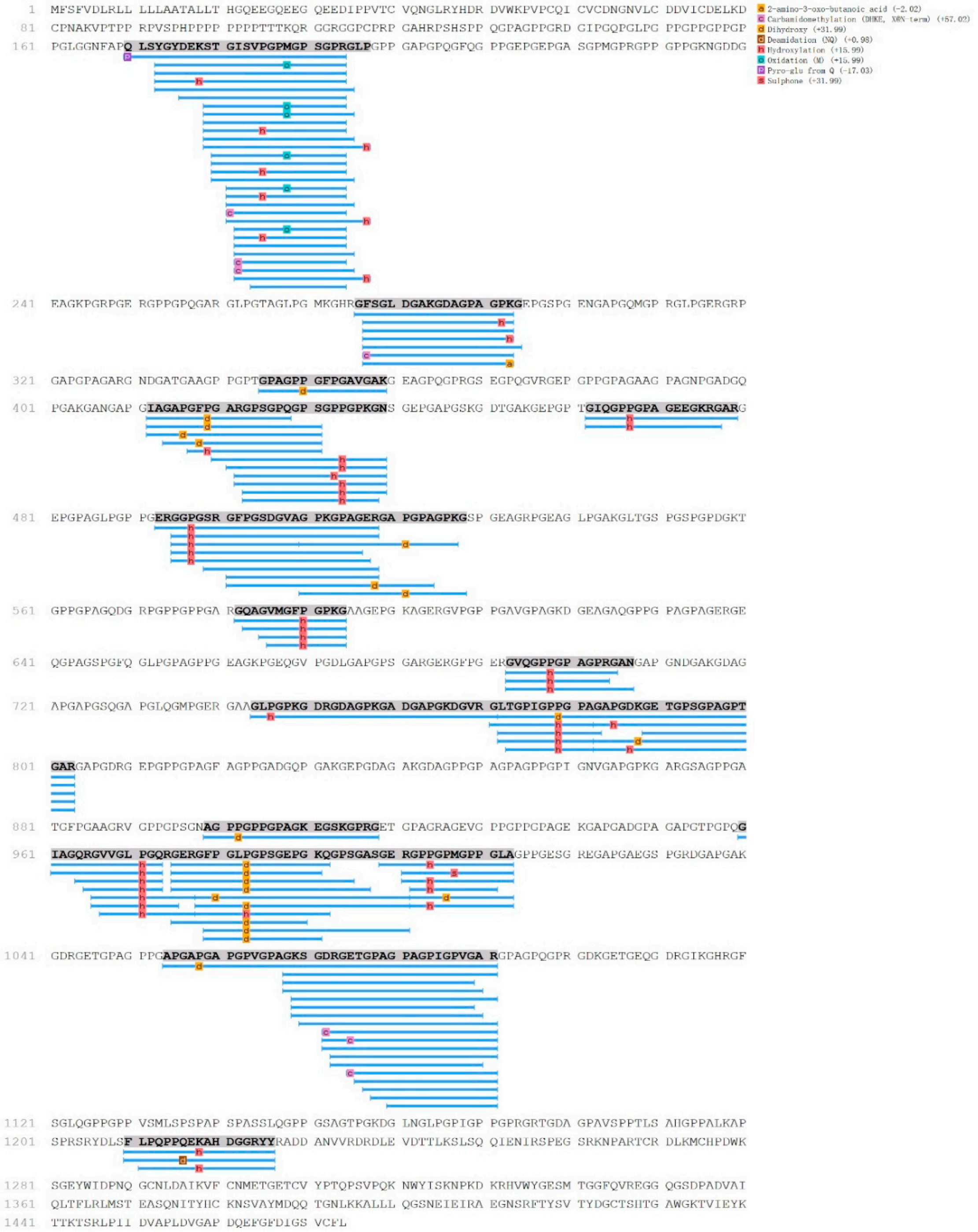

Figure S1. Sequence of collagen α-1(I) (tr|W5P481|W5P481_SHEEP Uncharacterized protein OS = Ovis aries GN = COL1A1 PE = 4 SV = 1) and the distribution of identified peptides.

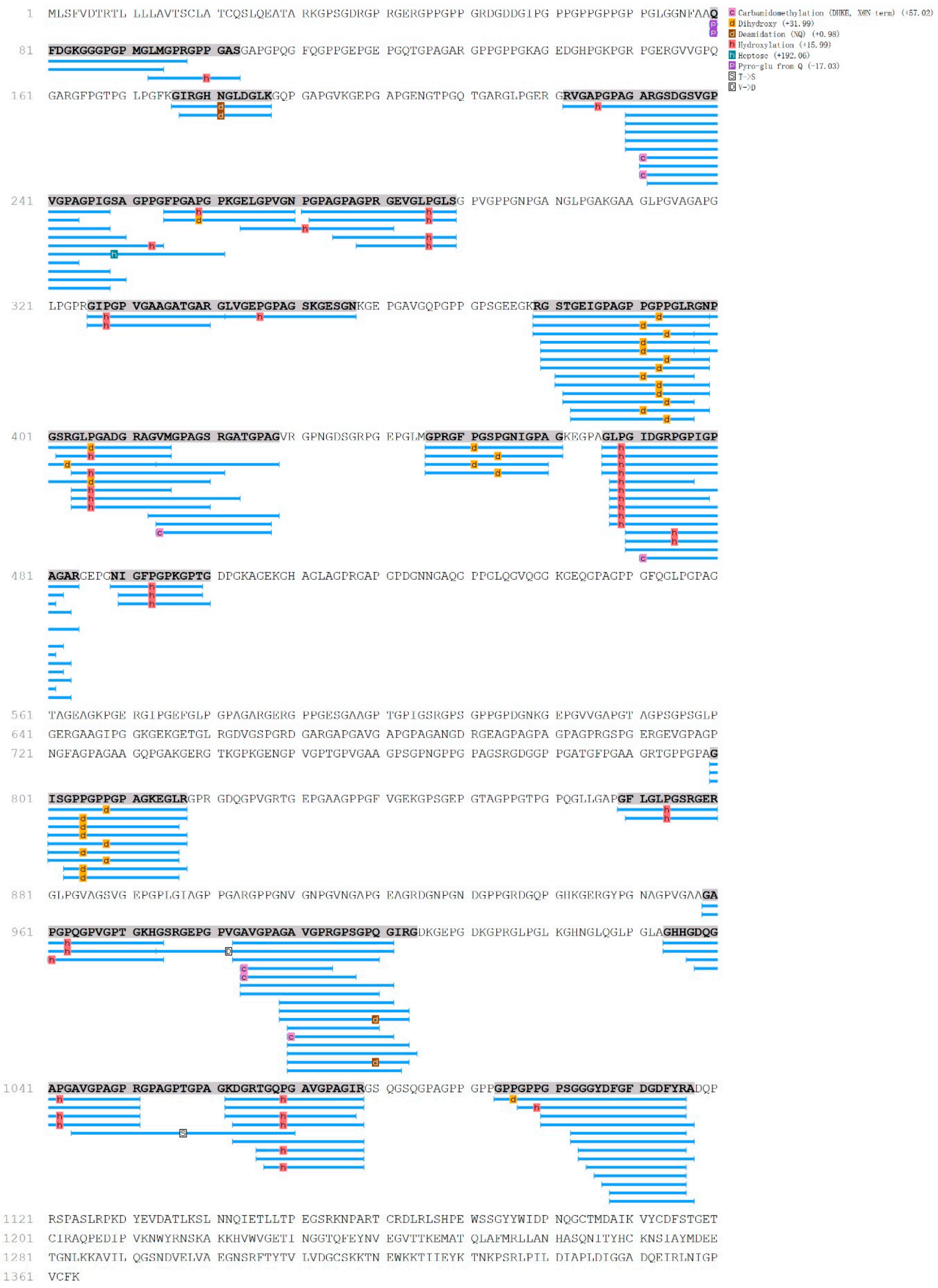

Figure S2. Sequence of collagen α-2(I) (tr|W5NTT7|W5NTT7_SHEEP Uncharacterized protein OS = Ovis aries GN = COL1A2 PE = 4 SV = 1) and the distribution of identified peptides.

Table S1. MS/MS ions of peptide LAGHHGDQGAPGAVGPAGPRGPAGPSGPAG.

#	b	b-H_2O	b-NH_3	B (2+)	Seq	y	y-H_2O	y-NH_3	y (2+)	#
1					L					30
2	185.13				A					29
3					G					28
4	379.21				H					27
5	516.27	498.26			H					26
6	573.29	555.28			G					25
7	688.32	670.30			D					24
8	816.37	798.36			Q					23
9	873.40	855.39	856.38		G					22
10	944.43	916.42	927.41		A					21
11	1057.48				P	1642.83				20
12	1114.49				G	1529.79				19
13	1185.54				A					18
14	1284.62	1266.61			V	1401.72				17
15	1341.61				G	1302.65		1285.62		16
16					P	1245.63	1227.63	1228.61	623.32	15
17					A	1148.58				14
18					G	1077.54	1059.53			13
19					P	1020.52	1002.51			12
20				910.46	R					11
21					G	767.37				10
22					P	710.34				9
23	2045.00				A					8
24	2102.02				G	542.25	524.24			7
25					P	485.24	467.22			6
26			2269.07		S	388.18				5
27	2343.14	2325.13			G	301.15	283.14			4
28					P	244.13	226.12			3
29					A	147.08				2
30					G					1

Table S2. Identified peptides generated from protein P02465.

	Peptides	Unique	-10lgP	Mass	Length	ppm	*m/z*	RT	Area	Scan
1	G.LAGHHGDQGAP(+15.99)GAVGPAGPRGPAGPSGPAG.K	1	27.61	2585.2483	30	0.9	862.7574	25.23	1.57×10^6	1098
2	N.RGEP(+15.99)GPAGAVGPAGAVGPRGPSGPQG.I	1	27.29	2268.1357	26	−0.1	757.0524	34.21	1.18×10^7	2196
3	A.GHHGDQGAP(+15.99)GAVGPAGPR.G	0	26.97	1652.7767	18	0	551.9329	15.45	3.15×10^6	320
4	A.GARGSDGSVGPVGPAGPIGSAGPP(+15.99)G.F	0	26.7	2089.0188	25	−0.5	1045.5161	44.84	5.02×10^6	3627
5	G.NRGEP(+15.99)GPAGAVGPAGAVGPRGPSGPQG.I	1	25.9	2382.1787	27	−0.4	795.0665	34	2.25×10^7	2167
6	V.GLP(+15.99)GIDGRPGPIGPAGA.R	0	25.4	1516.7997	17	0.1	759.4072	57.79	3.00×10^8	5329
7	N.PGPAGPAGPRGEVGLP(+15.99)GLS.G	0	25.27	1700.8845	19	−0.1	851.4495	54.9	9.17×10^6	4967
8	R.GPRGDQGPVGRSGETGASGPP(+15.99)G.F	1	25.23	2007.9358	22	−0.4	670.319	17.73	1.70×10^6	462
9	K.RGSTGEIGPAGPPGPP(+31.99)GLR.G	0	25.01	1803.9227	19	−0.3	902.9684	32.36	5.25×10^6	1953
10	G.LP(+15.99)GIDGRPGPIGPAG.A	0	24.36	1388.7411	15	0.3	695.3781	47.55	1.37×10^8	3976
11	A.GARGSDGSVGPVGPAGPIGSA.G	0	24.35	1764.8754	21	−0.5	883.4445	43.84	1.40×10^7	3491
12	G.LP(+15.99)GIDGRPGPIGPAGA.R	0	24.33	1459.7782	16	−1.1	730.8956	51.62	5.50×10^8	4525
13	A.GHHGDQGAP(+15.99)GAVGPAGPRGPAGPSGPAG.K	1	24.28	2401.127	28	0.6	801.3834	22.96	1.61×10^7	872
14	G.A(+57.02)RGSDGSVGPVGPAGPIGSA.G	0	24.21	1764.8754	20	−1	883.4441	44.26	1.40×10^7	3549
15	P.GPAGPAGPRGEVGLP(+15.99)GLS.G	0	24.01	1603.8318	18	−0.8	802.9225	52.32	1.35×10^7	4626
16	A.GHHGDQGAPGAVGPAGPRGPAGPSGPAG.K	1	23.8	2385.1321	28	−0.6	796.0508	24.83	3.88×10^6	1056
17	A.GAVGPAGAVGPRGPSGPQG.I	0	23.79	1587.8116	19	0.1	794.9131	32.01	2.48×10^7	1905
18	G.DQGAP(+15.99)GAVGPAGPRGPAGPSGPAG.K	1	23.61	2012.9663	24	−0.9	1007.4895	34.35	3.57×10^6	2213
19	G.AVGPRGPSGPQ(+.98)GIR.G	0	23.59	1348.7211	14	−0.3	450.5808	23.16	7.60×10^5	891
20	V.GLP(+15.99)GIDGRPGPIGPAG.A	0	23.56	1445.7626	16	0.3	723.8888	53.15	2.30×10^8	4741
21	G.LP(+15.99)GIDGRPGPIGPAGAR.G	0	23.54	1615.8794	17	0.7	539.6341	39.07	4.92×10^6	2820
22	Q.GAP(+15.99)GAVGPAGPRGPAGPSGPAG.K	1	23.52	1769.8809	22	−0.6	885.9472	29.51	4.72×10^6	1596
23	R.GSTGEIGPAGPP(+31.99)GPPGLR.G	0	23.51	1647.8215	18	−0.3	824.9178	39.49	1.41×10^8	2876
24	G.RVGAP(+15.99)GPAGARGSDGSVGPVGPAGPIG.S	0	23.5	2328.1934	27	0.4	777.072	42.56	1.30×10^7	3321
25	G.AVGPAGPRGPAGPSGPA.G	1	23.29	1414.7317	17	−0.1	708.373	26.72	7.98×10^6	1251
26	G.LP(+15.99)GIDGRPGPIGPA.G	0	23.12	1331.7197	14	0.5	666.8675	51.06	3.15×10^8	4447
27	A.GHHGDQGAP(+15.99)GAVGPAGPRGPAGPSGPA.G	1	23.08	2344.1057	27	−0.7	782.3753	23.45	2.00×10^6	919
28	A.GARGSDGSVGPVGPAGPIG.S	0	23.07	1606.8063	19	0.3	804.4106	42.37	5.30×10^7	3293
29	G.NRGEP(+15.99)GPAGAVGPAGAVGPRGPSGPQ.G	1	23.07	2325.1572	26	−0.8	776.0591	33.52	3.76×10^6	2104
30	G.SRGLP(+15.99)GADGRAGVM.G	0	22.91	1358.6725	14	−0.7	453.8978	27.35	7.41×10^6	1325
31	G.STGEIGPAGPP(+31.99)GPPGLR.G	0	22.73	1590.8002	17	−0.1	796.4073	39.9	5.59×10^7	2931
32	G.STGEIGPAGPPGP(+31.99)PGLRGN.P	0	22.71	1761.8645	19	−0.4	881.9391	39.06	1.49×10^7	2819
33	H.GNRGEP(+15.99)GPAGAVGPAGAVGPRGPSGPQG.I	1	22.63	2439.2002	28	0.8	814.0746	33.93	3.65×10^6	2158
34	G.IRGHN(+.98)GLDGLK.G	0	22.45	1179.636	11	−0.2	394.2192	18.73	4.44×10^6	538
35	G.AVGPAGPRGPAGPSGPAG.K	1	22.45	1471.7531	18	−0.1	736.8837	26.25	6.23×10^7	1202
36	V.GLP(+15.99)GIDGRPGPIGPA.G	0	22.29	1388.7411	15	−0.3	695.3776	54	3.04×10^8	4850
37	A.GAVGPAGAVGPRGPSGPQ.G	0	22.13	1530.7903	18	0.2	766.4026	31.23	4.46×10^7	1805
38	A.GAVGPRGPSGPQ(+.98)GIR.G	0	22	1405.7426	15	0.1	469.5882	23		876
39	G.AVGPAGAVGPRGPSGPQG.I	0	21.66	1530.7903	18	0.4	766.4027	29.8	4.46×10^7	1627
40	A.GHHGDQGAPGAVGPAGPR.G	0	21.58	1636.7817	18	0.9	546.6017	16.6	5.74×10^5	386

41	K.GIRGHN(+.98)GLDGLK.G	0	21.16	1236.6575	12	−0.4	413.2263	20.56	2.19×10^6	676
42	V.GLP(+15.99)GIDGRPGPIGPAGAR.G	0	21.14	1672.9009	18	0.1	558.6409	46.78	1.16×10^7	3891
43	G.FPGAP(+15.99)GPKGELGPVGN.P	0	21.01	1508.7623	16	−0.3	755.3882	45.31	2.18×10^7	3694
44	R.GSTGEIGPAGPPGP(+31.99)PGLRGN.P	0	21	1818.886	20	−0.1	910.4502	39.92	2.45×10^7	2936
45	G.AVGPRGPSGPQGIR.G	0	20.94	1347.7371	14	−0.8	450.2526	21.33	8.22×10^6	738
46	A.RGSDGSVGPVGPAGPIG.S	0	20.94	1478.7477	17	−0.8	740.3806	41.68		3193
47	A.VGPAGPRGPAGPSGPAG.K	1	20.93	1400.7159	17	−0.1	701.3652	25.84	4.20×10^6	1162
48	S.TGEIGPAGPPGP(+31.99)PGLRGN.P	0	20.89	1674.8325	18	−0.5	838.4232	39	3.20×10^6	2811
49	R.GSTGEIGPAGPPGPP(+31.99)GLRGN.P	0	20.78	1818.886	20	−0.1	910.4501	39.5	2.45×10^7	2878
50	H.GDQGAP(+15.99)GAVGPAGPR.G	0	20.67	1321.6375	15	0.2	661.8261	18.32	1.50×10^6	509
51	G.ARGSDGSVGPVGPAGPIG.S	0	20.62	1549.7848	18	0.1	775.8997	42.17	2.20×10^7	3264
52	S.GISGPP(+31.99)GPPGPAGKEGL.R	0	20.36	1518.7677	17	0.9	760.3918	35.85	5.46×10^6	2402
53	A.GVMGPAGSRGATGPAG.V	0	20.27	1341.6459	16	0.7	671.8307	23.94	1.88×10^6	966
54	V.GPAGPRGPAGPSGPAG.K	1	20.19	1301.6476	16	−0.8	651.8306	20.36		660
55	G.ELGPVGNP(+15.99)GPAGPAGPRG.E	0	19.8	1614.8114	18	−0.6	808.4125	35.08	4.83×10^6	2304
56	S.TGEIGPAGPPGPP(+31.99)GLR.G	0	19.78	1503.7681	16	−0.3	752.8911	39.42	1.52×10^7	2867
57	A.GAVGPRGPSGPQGIR.G	0	19.71	1404.7585	15	−0.7	469.2598	22.08	3.61×10^6	801
58	R.GIP(+15.99)GPVGAAGATGARG.L	0	19.66	1323.6895	16	0.3	662.8522	29.04	2.55×10^7	1537
59	A.GPAGPRGEVGLP(+15.99)GLS.G	0	19.61	1378.7205	15	0.9	690.3681	47.62	1.13×10^7	3989
60	G.AVGPAGAVGPRGPSGPQ.G	0	19.51	1473.7688	17	−0.7	737.8912	29.9	5.84×10^6	1638
61	S.GISGPPGPP(+31.99)GPAGKEGLR.G	0	19.47	1674.8689	18	−0.3	559.2968	25.75	4.01×10^7	1150
62	I.GQP(+15.99)GAVGPAGIR.G	0	19.44	1094.5833	12	0.1	548.299	22.73	2.36×10^6	852
63	G.A(+57.02)VGPRGPSGPQG.I	0	19.41	1135.5734	12	0.1	568.794	17.32	1.03×10^7	431
64	G.AVGPAGPRGPAGPS.G	1	19.27	1189.6204	14	−0.3	595.8173	21.68	1.21×10^6	768
65	P.GIDGRP(+15.99)GPIGPAGA.R	0	19.15	1249.6415	14	−0.1	625.8279	30.9	1.88×10^6	1764
66	G.I(+57.02)DGRPGPIGPAGA.R	0	19.07	1233.6465	13	0.3	617.8307	36.48	5.06×10^6	2486
67	A.GAVGPRGPSGPQG.I	0	19.07	1135.5734	13	0.1	568.794	18.46	1.03×10^7	518
68	G.A(+57.02)VGPAGAVGPRGPS.G	0	18.9	1248.6575	14	−0.8	625.3355	27.2	3.39×10^6	1305
69	G.LMGPRGPP(+15.99)GAS.G	0	18.84	1054.5229	11	0.2	528.2689	22.6	7.21×10^5	844
70	K.RGSTGEIGPAGPP(+31.99)GPPGLRGN.P	0	18.8	1974.9871	21	0	659.3363	32.42	4.49×10^7	1959
71	G.ISGPPGPP(+31.99)GPAGKEGLR.G	0	18.66	1617.8474	17	0.1	809.931	26.74	8.22×10^6	1254
72	R.GSTGEIGPAGPP(+31.99)GPPGLRGN.P	0	18.66	1818.886	20	0.2	910.4504	38.11	2.45×10^7	2690
73	R.GNPGSRGLP(+31.99)GADGRAGVMGPAGS.R	0	18.63	2068.9707	23	0.3	690.6644	34.91	4.52×10^6	2283
74	A.GARGSDGSVGPVGPA.G	0	18.46	1282.6265	15	0.2	642.3206	23.02	3.26×10^6	878
75	P.VGS(sub L)PGIDGRPGPIGPAGA.R	1	18.46	1573.8212	18	0.1	787.9179	56.01	8.09×10^5	5114
76	G.AVGPRGPSGPQGI.R	0	18.46	1191.636	13	−0.4	596.825	30.76	9.34×10^5	1747
77	K.RGSTGEIGPAGPPGP(+31.99)PGLRGN.P	0	18.42	1974.9871	21	0.1	659.3364	30.91	4.49×10^7	1766
78	R.GLP(+15.99)GADGRAGVMGPAGSRGA.T	0	18.4	1768.8638	20	0.4	590.6288	33.4	6.17×10^6	2090
79	G.V(+57.02)MGPAGSRGATGPA.G	0	18.2	1284.6244	14	0	643.3195	24.23		999
80	S.GPP(+31.99)GPPGPAGKEGLR.G	0	18.13	1417.7313	15	0.1	473.5844	19.63	2.94×10^7	600
81	G.ISGPPGPP(+31.99)GPAGKEGL.R	0	17.98	1461.7463	16	−0.1	731.8804	36	2.18×10^7	2424
82	G.A(+57.02)RGSDGSVGPVGPA.G	0	17.93	1282.6266	14	−0.3	642.3204	22.2	3.26×10^6	811

83	G.FPGAP(+31.99)GPKGELGPVGN.P	0	17.85	1524.7572	16	0.4	763.3862	45.64	2.89×10^{6}	3739
84	N.PGSRGLP(+31.99)GADGRAGVM.G	0	17.6	1528.7416	16	0.3	510.588	27.83	7.73×10^{6}	1384
85	G.DQGAP(+15.99)GAVGPAGPR.G	0	17.59	1264.616	14	0.4	633.3155	19.25	8.49×10^{5}	576
86	S.GISGPP(+31.99)GPPGPAGKEGLR.G	0	17.52	1674.8689	18	0.7	559.2973	28.74	9.43×10^{7}	1501
87	G.LP(+15.99)GIDGRPGPIG.P	0	17.48	1163.6298	12	0.1	582.8222	48.07	4.57×10^{6}	4050
88	R.GIP(+15.99)GPVGAAGATGAR.G	0	17.47	1266.668	15	0	634.3412	28.9	4.42×10^{6}	1521
89	G.LVGEP(+15.99)GPAGSKGESGN.K	0	17.4	1470.6949	16	1.2	736.3557	17.62	3.58×10^{5}	453
90	T.GEIGPAGPP(+31.99)GPPGLRGN.P	0	17.39	1573.7848	17	0	787.8997	38.45	1.37×10^{6}	2735
91	T.GEIGPAGPPGPP(+31.99)GLR.G	0	17.38	1402.7205	15	0.4	702.3678	39.29	4.76×10^{7}	2849
92	R.GLP(+15.99)GADGRAGVMGPAGS.R	0	17.17	1484.7041	17	−1	743.3586	41.88	6.34×10^{6}	3223
93	G.ISGPP(+31.99)GPPGPAGKEGLR.G	0	17.11	1617.8474	17	0.9	540.2902	25.74	1.58×10^{8}	1148
94	A.GPRGEVGLP(+15.99)GLS.G	0	17.07	1153.6091	12	0.8	577.8123	42.11	3.56×10^{7}	3255
95	G.AVGPRGPSGPQGIRG.D	0	16.94	1404.7585	15	−0.4	469.2599	22.53	3.61×10^{6}	838
96	G.ISGPP(+31.99)GPPGPAGKEGL.R	0	16.87	1461.7463	16	1.2	731.8813	35.65	2.18×10^{7}	2377
97	G.VMGPAGSRGATGPAG.V	0	16.82	1284.6244	15	0.7	643.3199	21.31	2.21×10^{6}	736
98	M.GPRGFPGSP(+31.99)GNIGPA.G	0	16.77	1411.6843	15	0.2	706.8495	36.48	5.07×10^{7}	2487
99	R.GPRGDQGPVGRSGETGASGPPGFS(sub V).G	1	16.73	2226.0413	24	−1	743.0203	34.01	3.17×10^{7}	2168
100	R.GLP(+15.99)GADGRAGVM.G	0	16.66	1115.5393	12	0.1	558.777	34.78	4.24×10^{7}	2267
101	G.AVGPRGPSGPQ.G	0	16.59	1021.5305	11	−0.3	511.7724	16.92	2.85×10^{6}	405
102	G.AVGPAGPRGPAGPSGPAGKDGR.I	1	16.46	1927.9976	22	0.3	483.0068	19.57	6.06×10^{5}	596
103	G.FLGLP(+15.99)GSRGER.G	0	16.42	1203.636	11	0.2	602.8254	33.39	4.72×10^{6}	2088
104	R.GNPGSR(+31.99)GLPGADGRAG.V	0	16.2	1469.697	16	0.4	735.8561	17.26	5.32×10^{5}	426
105	S.GPP(+31.99)GPPGPAGKEGL.R	0	16.11	1261.6302	14	0.2	631.8225	26.16	7.44×10^{6}	1194
106	G.LP(+15.99)GIDGRPGP.I	0	16.09	993.5243	10	−0.1	497.7694	37.02	6.23×10^{6}	2554
107	G.VMGPAGSRGATGPA.G	0	16.08	1227.603	14	−1.8	614.8077	21	6.94×10^{5}	712
108	E.FGFDGDFYRA.D	0	15.97	1193.5142	10	0.1	597.7644	75.78	2.26×10^{7}	7183
109	E.FGFDGDFYR.A	0	15.89	1122.4771	9	0.4	562.246	67.98	1.05×10^{7}	6483
110	M.GPRGFP(+31.99)GSPGNIGPA.G	0	15.88	1411.6843	15	−0.1	706.8494	34.49	5.07×10^{7}	2231
111	Q.GLPGLAGHHGDQGAP(+31.99)GAVGPAGPRGPAGPSGPAG.K	1	15.6	2925.4229	34	0	976.1482	38.6	1.62×10^{7}	2755
112	P.GFLGLP(+15.99)GSRGER.G	0	15.56	1260.6575	12	−0.3	421.2263	35.18	1.75×10^{6}	2316
113	G.A(+57.02)VGPAGAVGPR.G	0	15.52	1007.5512	11	0.5	504.7831	22.78	6.94×10^{5}	857
114	M.GPRGFPGSP(+31.99)GNIGPAG.K	0	15.45	1468.7058	16	0.1	735.3602	34.83	1.96×10^{8}	2273
115	M.GPRGFP(+31.99)GSPGNIGPAG.K	0	15.37	1468.7058	16	0.2	735.3604	32.68	1.96×10^{8}	1995
116	A.GARGSDGSVGPVGPAGPIGS(+192.06)AGPPGFPGAPGP.K	0	15.29	2888.394	32	2.7	963.8079	55.49		5045
117	Q.GLP(+31.99)GLAGHHGDQGAPGAVGPAGPRGPAGPSGPAG.K	1	15.19	2925.4229	34	−0.2	976.1481	38.95	1.62×10^{7}	2805
118	P.GIDGRP(+15.99)GPIGPAG.A	0	15.18	1178.6044	13	0.1	590.3095	27.06	9.47×10^{5}	1287
119	R.GLP(+15.99)GADGRAGVMGPAGSR.G	0	15.16	1640.8052	18	2.3	547.9436	31.4	2.02×10^{7}	1827
120	P.GIDGRPGPIGPA.G	0	15.01	1105.5879	12	1.4	553.802	34.27	1.20×10^{6}	2204

Table S3. All peptides identified from *Elaphuri Davidiani Cornu* fraction

	Peptides	−10lgP	Mass	Length	ppm	m/z	RT	Area	GRAVY
1	G.APGAP(+31.99)GAPGPVGPAGKSGDRGETGPAGPAGPIGPVGAR.G	28.56	3288.6599	38	−0.7	823.1717	41.39	1.14×10^7	−0.397
2	Q.GLPGLAGHHGDQGAP(+31.99)GAVGPAGPRGPAGPSGPAG.K	15.6	2925.4229	34	0	976.1482	38.6	1.62×10^7	−0.356
3	Q.GLP(+31.99)GLAGHHGDQGAPGAVGPAGPRGPAGPSGPAG.K	15.19	2925.4229	34	−0.2	976.1481	38.95	1.62×10^7	−0.356
4	A.GARGSDGSVGPVGPAGPIGS(+192.06)AGPPGFPGAPGP.K	15.29	2888.394	32	2.7	963.8079	55.49		−0.147
5	G.LTGPIGPP(+31.99)GPAGAPGDKGETGPSGPAGPTGAR.G	21.52	2825.3943	32	−0.3	942.8051	39.24	4.06×10^7	−0.613
6	G.LAGHHGDQGAP(+15.99)GAVGPAGPRGPAGPSGPAG.K	27.61	2585.2483	30	0.9	862.7574	25.23	1.57×10^6	−0.45
7	A.GLP(+15.99)GPKGDRGDAGPKGADGAPGKDGVRG.L	17.43	2574.2898	28	−0.4	644.5795	18.96	3.95×10^6	−1.132
8	P.Q(−17.03)LSYGYDEKSTGISVPGPMGPSGPR.G	30.85	2562.2061	25	0.9	1282.112	77.11	3.26×10^6	−0.776
9	G.ERGGP(+15.99)GSRGFPGSDGVAGPKGPAGER.G	19.03	2467.1951	26	1.9	617.8072	23.43	1.07×10^6	−1.112
10	H.GSRGEPGPD(sub V)GAVGPAGAVGPRGPSGPQG.I	18.69	2440.1843	28	−0.8	814.4014	35.92	2.63×10^6	−0.656
11	H.GNRGEP(+15.99)GPAGAVGPAGAVGPRGPSGPQG.I	22.63	2439.2002	28	0.8	814.0746	33.93	3.65×10^6	−0.654
12	A.GHHGDQGAP(+15.99)GAVGPAGPRGPAGPSGPAG.K	24.28	2401.127	28	0.6	801.3834	22.96	1.61×10^7	−0.682
13	A.GHHGDQGAPGAVGPAGPRGPAGPSGPAG.K	23.8	2385.1321	28	−0.6	796.0508	24.83	3.88×10^6	−0.682
14	G.NRGEP(+15.99)GPAGAVGPAGAVGPRGPSGPQG.I	25.9	2382.1787	27	−0.4	795.0665	34	2.25×10^7	−0.663
15	G.AVGPAGPRGPAGPS(sub T)GPAGKDGRTGQPG.A	20.62	2368.1995	27	0.2	790.4072	22.39	4.69×10^6	−0.91
16	P.GPP(+31.99)GPPGPSGGGYDFGFDGDFYR.A	18.65	2346.9817	23	1.6	783.3358	83.42	3.07×10^6	−0.922
17	A.GHHGDQGAP(+15.99)GAVGPAGPRGPAGPSGPA.G	23.08	2344.1057	27	−0.7	782.3753	23.45	2.00×10^6	−0.693
18	G.RVGAP(+15.99)GPAGARGSDGSVGPVGPAGPIG.S	23.5	2328.1934	27	0.4	777.072	42.56	1.30×10^7	−0.052
19	G.NRGEP(+15.99)GPAGAVGPAGAVGPRGPSGPQ.G	23.07	2325.1572	26	−0.8	776.0591	33.52	3.76×10^6	−0.673
20	S.YGYDEKSTGISVPGPMGPSGPRG.L	20.55	2308.0793	23	−0.4	770.3668	56.93	5.18×10^6	−0.839
21	N.RGEP(+15.99)GPAGAVGPAGAVGPRGPSGPQG.I	27.29	2268.1357	26	−0.1	757.0524	34.21	1.18×10^7	−0.554
22	S.YGYDEK(+15.99)STGISVPGPMGPSGPR.G	27.07	2267.0527	22	0.8	756.6921	56.68	3.36×10^7	−0.859
23	S.YGYDEKSTGISVPGPM(+15.99)GPSGPR.G	20.03	2267.0527	22	−0.4	756.6912	49.46	1.29×10^7	−0.859
24	R.GFP(+31.99)GLPGPSGEPGKQGPSGASGER.G	18.91	2254.0613	24	−0.9	752.3604	38.43	4.22×10^7	−1.004
25	R.GFPGLP(+31.99)GPSGEPGKQGPSGASGER.G	18.45	2254.0613	24	−0.9	752.3604	38.1	4.22×10^7	−1.004
26	S.YGYDEKSTGISVPGPMGPSGPR.G	24.63	2251.0579	22	0.4	751.3602	56.72	5.77×10^7	−0.859
27	R.GPRGDQGPVGRSGETGASGPPGFS(sub V).G	16.73	2226.0413	24	−1	743.0203	34.01	3.17×10^7	−0.839
28	G.FPGLP(+31.99)GPSGEPGKQGPSGASGER.G	21.8	2197.0398	23	1.2	733.3547	37.17	7.61×10^6	−1.03
29	R.GGP(+15.99)GSRGFPGSDGVAGPKGPAGER.G	25.13	2182.0515	24	−0.1	728.3577	26.27	5.78×10^6	−0.871
30	R.GERGFPGLP(+31.99)GPSGEPGKQGPSGA.S	19.72	2167.0293	23	0.8	723.351	40.08	3.43×10^7	−1.013
31	A.GKSGDRGETGPAGPAGPIGPVGAR.G	25.45	2160.1035	24	0.4	721.0421	27.86	2.20×10^6	−0.704
32	R.GFPGSDGVAGPKGPAGER(+31.99)GAPGPA.G	15.78	2137.0188	24	−0.4	713.3466	34.37	5.74×10^6	−0.55
33	G.KSGDRGETGPAGPAGPIGPVGAR.G	27.98	2103.082	23	0.3	702.0349	29.54	1.21×10^7	−0.717
34	A.GARGSDGSVGPVGPAGPIGSAGPP(+15.99)G.F	26.7	2089.0188	25	−0.5	1045.516	44.84	5.02×10^6	−0.148
35	P.GPP(+15.99)GPSGGGYDFGFDGDFYR.A	24.34	2079.8599	20	−0.2	1040.937	83.03	1.54×10^6	−0.88
36	R.GNPGSRGLP(+31.99)GADGRAGVMGPAGS.R	18.63	2068.9707	23	0.3	690.6644	34.91	4.52×10^6	−0.448
37	R.GERGFPGLP(+31.99)GPSGEPGKQGPS.G	18.89	2038.9707	21	−0.3	680.664	37.68	7.73×10^6	−1.176
38	R.GGP(+15.99)GSRGFPGSDGVAGPKGPAGE.R	21.63	2025.9503	23	1.1	676.3248	31.92		−0.713
39	S.FLPQPPQEK(+15.99)AHDGGRYY.R	16.17	2017.9646	17	0	673.6621	41.86	9.29×10^7	−1.494
40	G.DQGAP(+15.99)GAVGPAGPRGPAGPSGPAG.K	23.61	2012.9663	24	−0.9	1007.49	34.35	3.57×10^6	−0.496

41	R.GPRGDQGPVGRSGETGASGPP(+15.99)G.F	25.23	2007.9358	22	−0.4	670.319	17.73	1.70×10^6	−1.155
42	A.GKSGDRGETGPAGPAGPIGPVGA.R	20.39	2004.0024	23	0.4	669.0084	37.67	4.43×10^6	−0.539
43	S.FLPQPPQ(+.98)EKAHDGGRYY.R	15.12	2002.9536	17	−0.1	501.7456	43.96	1.29×10^7	−1.494
44	K.RGSTGEIGPAGPP(+31.99)GPPGLRGN.P	18.8	1974.9871	21	0	659.3363	32.42	4.49×10^7	−0.867
45	K.RGSTGEIGPAGPPGP(+31.99)PGLRGN.P	18.42	1974.9871	21	0.1	659.3364	30.91	4.49×10^7	−0.867
46	K.SGDRGETGPAGPAGPIGPVGAR.G	25.37	1974.9871	22	−0.3	659.3361	34.5	4.49×10^7	−0.573
47	G.KSGDRGETGPAGPAGPIGPVGA.R	21.33	1946.981	22	0.2	650.001	37.72	3.08×10^7	−0.545
48	A.GKSGDRGETGPAGPAGPIGPVG.A	15.21	1932.9653	22	−0.6	645.3287	33.63	3.13×10^6	−0.645
49	G.AVGPAGPRGPAGPSGPAGKDGR.I	16.46	1927.9976	22	0.3	483.0068	19.57	6.06×10^5	−0.755
50	G.SRGFPGSDGVAGPKGPAGER.G	24.74	1897.9393	20	−0.2	633.6536	25.46	9.77×10^6	−0.905
51	R.GGP(+15.99)GSRGFPGSDGVAGPKGPAG.E	16.06	1896.9077	22	−0.1	633.3098	30.58	2.38×10^6	−0.586
52	G.D(+57.02)RGETGPAGPAGPIGPVGAR.G	26.14	1887.9551	20	0	630.3256	36.16	6.15×10^6	−0.57
53	G.DRGE(+57.02)TGPAGPAGPIGPVGAR.G	25.81	1887.9551	20	−0.3	630.3254	34.43	1.54×10^7	−0.57
54	P.GPSGGGYDFGFDGDFYRA.D	24.13	1883.775	18	0.2	942.895	86.18	3.60×10^6	−0.678
55	G.KSGDRGETGPAGPAGPIGPVG.A	19.5	1875.9438	21	0.3	626.3221	36.04	6.20×10^6	−0.657
56	F.LPQPPQEK(+15.99)AHDGGRYY.R	16.39	1870.8961	16	0.2	468.7314	31.68	1.08×10^7	−1.762
57	Y.DEKSTGISVPGPMGPSGPR.G	23.23	1867.9098	19	0.6	623.6442	44.79	2.41×10^7	−0.837
58	A.GAPGDK(+31.99)GETGPSGPAGPTGAR.G	17.75	1867.866	21	−0.1	623.6292	17.33	6.61×10^5	−1.019
59	K.SGLGSQVGLM(+15.99)PGSVGPVGPR.G	21.77	1866.9622	20	0.8	934.4891	60.6	1.44×10^6	0.225
60	A.GAPGD(+15.99)KGETGPSGPAGPTGAR.G	24.65	1851.8711	21	0.3	618.2979	17.43	3.87×10^6	−1.019
61	A.GAP(+15.99)GDKGETGPSGPAGPTGAR.G	21.84	1851.8711	21	0.1	618.2977	17.08	3.87×10^6	−1.019
62	G.DRGETGPAGPAGPIGPVGAR.G	24.3	1830.9336	20	−0.4	611.3182	38.57	6.41×10^6	−0.57
63	R.GSTGEIGPAGPPGP(+31.99)PGLRGN.P	21	1818.886	20	−0.1	910.4502	39.92	2.45×10^7	−0.685
64	R.GSTGEIGPAGPPGPP(+31.99)GLRGN.P	20.78	1818.886	20	−0.1	910.4501	39.5	2.45×10^7	−0.685
65	R.GSTGEIGPAGPP(+31.99)GPPGLRGN.P	18.66	1818.886	20	0.2	910.4504	38.11	2.45×10^7	−0.685
66	P.GPSGGGYDFGFDGDFYR.A	25.82	1812.7379	17	−0.1	907.3762	83.86	7.32×10^6	−0.824
67	G.IAGAPGFP(+31.99)GARGPSGPQGPS.G	19.47	1808.8805	20	−0.1	905.4474	39.39	1.38×10^7	−0.365
68	G.IAGAP(+31.99)GFPGARGPSGPQGPS.G	17.69	1808.8805	20	−0.1	905.4474	39.03	1.38×10^7	−0.365
69	K.RGSTGEIGPAGPPGPP(+31.99)GLR.G	25.01	1803.9227	19	−0.3	902.9684	32.36	5.25×10^6	−0.753
70	N.AGPP(+31.99)GPPGPAGKEGSKGPRG.E	16.95	1801.907	20	−0.4	451.4838	15.5		−1.27
71	R.GERGFPGLP(+31.99)GPSGEPGKQ.G	15.65	1797.8645	18	0	600.2955	34.28		−1.217
72	P.GARGPSGPQGPSGPP(+15.99)GPKGN.S	23.28	1786.871	20	−0.5	596.6306	17.12	1.20×10^6	−1.38
73	K.STGISVPGPMGPSGPRGLP(+15.99).G	22.13	1778.8984	19	−0.4	890.4562	62.14	2.74×10^6	−0.168
74	Q.GAP(+15.99)GAVGPAGPRGPAGPSGPAG.K	23.52	1769.8809	22	−0.6	885.9472	29.51	4.72×10^6	−0.223
75	R.GLP(+15.99)GADGRAGVMGPAGSRGA.T	18.4	1768.8638	20	0.4	590.6288	33.4	6.17×10^6	−0.11
76	A.GARGSDGSVGPVGPAGPIGSA.G	24.35	1764.8754	21	−0.5	883.4445	43.84	1.40×10^7	0.014
77	G.A(+57.02)RGSDGSVGPVGPAGPIGSA.G	24.21	1764.8754	20	−1	883.4441	44.26	1.40×10^7	0.035
78	G.STGEIGPAGPPGP(+31.99)PGLRGN.P	22.71	1761.8645	19	−0.4	881.9391	39.06	1.49×10^7	−0.7
79	T.GIQGPP(+15.99)GPAGEEGKRGAR.G	19.12	1748.8917	18	0.2	438.2303	17.22	6.09×10^5	−1.25
80	A.Q(−17.03)FDGKGGGPGPMGLMGPR.G	21.29	1740.8075	18	1.7	871.4125	75.71	1.42×10^6	−0.7
81	G.ARGPSGPQGPSGPP(+15.99)GPKGN.S	20.95	1729.8495	19	0.6	577.6241	17.04		−1.432
82	D.RGETGPAGPAGPIGPVGAR.G	21.78	1715.9066	19	0.4	572.9764	31.77	1.03×10^7	−0.416

83	N.PGPAGPAGPRGEVGLP(+15.99)GLS.G	25.27	1700.8845	19	−0.1	851.4495	54.9	9.17×10^{6}	−0.2
84	A.GPKGPAGERGAP(+31.99)GPAGPKG.S	17.4	1688.8594	19	−0.5	563.9601	15.31	6.01×10^{4}	−1.116
85	S.GISGPPGPP(+31.99)GPAGKEGLR.G	19.47	1674.8689	18	−0.3	559.2968	25.75	4.01×10^{7}	−0.722
86	S.GISGPP(+31.99)GPPGPAGKEGLR.G	17.52	1674.8689	18	0.7	559.2973	28.74	9.43×10^{7}	−0.722
87	S.TGEIGPAGPPGP(+31.99)PGLRGN.P	20.89	1674.8325	18	−0.5	838.4232	39	3.20×10^{6}	−0.694
88	A.GLP(+15.99)GIDGRPGPIGPAGAR.G	21.14	1672.9009	18	0.1	558.6409	46.78	1.16×10^{7}	−0.272
89	R.GERGFPGLP(+31.99)GPSGEPGK.Q	18.35	1669.8059	17	0.1	557.6093	35.56	3.67×10^{7}	−1.082
90	A.RGPSGPQGPSGP(+15.99)PGPKGN.S	23.74	1658.8124	18	−0.1	553.9447	15.67	7.75×10^{6}	−1.611
91	A.RGPSGPQGPSGPP(+15.99)GPKGN.S	23.49	1658.8124	18	−0.5	553.9445	16.91	7.75×10^{6}	−1.611
92	R.GFPGSDGVAGPKGPAGER.G	16.2	1654.8063	18	0.1	552.6094	31.75	7.40×10^{7}	−0.711
93	A.GHHGDQGAP(+15.99)GAVGPAGPR.G	26.97	1652.7767	18	0	551.9329	15.45	3.15×10^{6}	−0.861
94	G.KDGRTGQP(+15.99)GAVGPAGIR.G	24.45	1651.8754	17	0.5	551.6327	18.07	4.42×10^{6}	−0.794
95	R.GSTGEIGPAGPP(+31.99)GPPGLR.G	23.51	1647.8215	18	−0.3	824.9178	39.49	1.41×10^{8}	−0.544
96	S.GGGYDFGFDGDFYRA.D	19.62	1642.6687	15	0	822.3416	87.72	3.46×10^{7}	−0.627
97	R.GLP(+15.99)GADGRAGVMGPAGSR.G	15.16	1640.8052	18	2.3	547.9436	31.4	2.02×10^{7}	−0.2
98	A.GHHGDQGAPGAVGPAGPR.G	21.58	1636.7817	18	0.9	546.6017	16.6	5.74×10^{5}	−0.861
99	G.KDGRTGQPGAVGPAGIR.G	23	1635.8805	17	−0.1	546.3007	20.27	1.67×10^{6}	−0.794
100	A.GPKGPAGERGAP(+31.99)GPAGPK.G	16.93	1631.8379	18	0.4	544.9534	15.2		−1.156
101	A.GAPGF(+31.99)PGARGPSGPQGPS.G	18.51	1624.7593	18	−0.1	813.3868	31.32	9.92×10^{6}	−0.756
102	G.ISGPPGPP(+31.99)GPAGKEGLR.G	18.66	1617.8474	17	0.1	809.931	26.74	8.22×10^{6}	−0.741
103	G.ISGPP(+31.99)GPPGPAGKEGLR.G	17.11	1617.8474	17	0.9	540.2902	25.74	1.58×10^{8}	−0.741
104	G.LP(+15.99)GIDGRPGPIGPAGAR.G	23.54	1615.8794	17	0.7	539.6341	39.07	4.92×10^{6}	−0.265
105	G.ELGPVGNP(+15.99)GPAGPAGPRG.E	19.8	1614.8114	18	−0.6	808.4125	35.08	4.83×10^{6}	−0.572
106	A.GARGSDGSVGPVGPAGPIG.S	23.07	1606.8063	19	0.3	804.4106	42.37	5.30×10^{7}	−0.037
107	P.GPAGPAGPRGEVGLP(+15.99)GLS.G	24.01	1603.8318	18	−0.8	802.9225	52.32	1.35×10^{7}	−0.122
108	G.F(+57.02)SGLDGAKGDAGPAGPK.G	22.45	1600.7844	17	−0.5	534.6018	35.6	2.59×10^{6}	−0.518
109	R.GFSGLDGAKGDAGPAGPK.G	19.32	1600.7844	18	0.5	534.6024	33.61	6.62×10^{6}	−0.511
110	G.FSGLDGAKGDAGPAGPKG.E	17.08	1600.7844	18	0.9	534.6025	30.48	6.05×10^{6}	−0.511
111	T.GISVPGPMGPSGPRGLP(+15.99).G	19.34	1590.8187	17	0.3	796.4169	60.83	3.86×10^{6}	−0.1
112	G.STGEIGPAGPP(+31.99)GPPGLR.G	22.73	1590.8002	17	−0.1	796.4073	39.9	5.59×10^{7}	−0.553
113	V.GAVGPAGAVGPRGPSGPQG.I	23.79	1587.8116	19	0.1	794.9131	32.01	2.48×10^{7}	−0.221
114	G.GGYDFGFDGDFYRA.D	17.51	1585.6473	14	0.2	793.8311	88.25	2.39×10^{6}	−0.643
115	P.VGS(sub L)PGIDGRPGPIGPAGA.R	18.46	1573.8212	18	0.1	787.9179	56.01	8.09×10^{5}	−0.059
116	T.GEIGPAGPP(+31.99)GPPGLRGN.P	17.39	1573.7848	17	0	787.8997	38.45	1.37×10^{6}	−0.694
117	S.GGGYDFGFDGDFYR.A	21.2	1571.6316	14	0.1	786.8231	84.56	6.03×10^{7}	−0.8
118	K.STGISVPGPM(+15.99)GPSGPRG.L	16.25	1568.7616	17	−0.2	785.3879	38.12	4.79×10^{6}	−0.318
119	G.IAGAPGFP(+31.99)GARGPSGPQ.G	17.73	1567.7742	17	0.6	784.8948	36.03	1.11×10^{7}	−0.265
120	G.E(+57.02)TGPAGPAGPIGPVGAR.G	24.14	1559.8055	17	−0.4	780.9097	39.5	1.86×10^{7}	−0.176
121	D.RGETGPAGPAGPIGPVGA.R	20.31	1559.8055	18	−0.3	780.9099	41.78	4.14×10^{6}	−0.189
122	G.FSGLDGAKGDAGPAGPK(+15.99).G	17.59	1559.7579	17	0.7	520.9269	27.58	7.70×10^{6}	−0.518
123	G.FSGLDGAKGDAGPAGP(+15.99)K.G	16.59	1559.7579	17	0.4	520.9268	26.79	7.70×10^{6}	−0.518
124	K.STGISVPGPMGPSGPRG.L	19.2	1552.7667	17	−0.4	777.3903	47.28	3.65×10^{7}	−0.318

125	G.ARGSDGSVGPVGPAGPIG.S	20.62	1549.7848	18	0.1	775.8997	42.17	2.20×10^7	−0.017
126	G.FSGLDGAKGDAGPAGPK.G	25.11	1543.7629	17	−0.7	772.8882	29.74	2.36×10^6	−0.518
127	G.FSGLDGAKGDAGPAGPK(−2.02).G	21.96	1541.7473	17	−0.6	771.8805	37.82	3.10×10^6	−0.518
128	R.GERGFPGLP(+31.99)GPSGEPG.K	16.77	1541.7109	16	0.2	771.8629	45.13	3.35×10^7	−0.906
129	G.ISVPGPMGPSGPRGLP(+15.99).G	20.77	1533.7972	16	−0.3	767.9057	58.83	3.46×10^6	−0.081
130	A.GAVGPAGAVGPRGPSGPQ.G	22.13	1530.7903	18	0.2	766.4026	31.23	4.46×10^7	−0.211
131	G.AVGPAGAVGPRGPSGPQG.I	21.66	1530.7903	18	0.4	766.4027	29.8	4.46×10^7	−0.211
132	N.PGSRGLP(+31.99)GADGRAGVM.G	17.6	1528.7416	16	0.3	510.588	27.83	7.73×10^6	−0.312
133	G.FPGAP(+31.99)GPKGELGPVGN.P	17.85	1524.7572	16	0.4	763.3862	45.64	2.89×10^6	−0.419
134	K.DGRTGQP(+15.99)GAVGPAGIR.G	25.55	1523.7804	16	0.4	508.9343	23.6	8.01×10^6	−0.6
135	T.GIQGPP(+15.99)GPAGEEGKRG.A	19.19	1521.7535	16	0.2	508.2586	18.18	9.25×10^5	−1.238
136	A.GISGPP(+31.99)GPPGPAGKEGL.R	20.36	1518.7677	17	0.9	760.3918	35.85	5.46×10^6	−0.5
137	V.GLP(+15.99)GIDGRPGPIGPAGA.R	25.4	1516.7997	17	0.1	759.4072	57.79	3.00×10^8	−0.024
138	G.GGYDFGFDGDFYR.A	20.74	1514.6102	13	−0.3	758.3121	83.11	4.14×10^6	−0.831
139	K.STGISVP(+15.99)GPMGPSGPR.G	21.93	1511.7402	16	0.3	756.8776	41.76	4.07×10^7	−0.312
140	K.STGISVPGPM(+15.99)GPSGPR.G	18.97	1511.7402	16	−0.2	756.8773	38.04	5.56×10^7	−0.312
141	G.FPGAP(+15.99)GPKGELGPVGN.P	21.01	1508.7623	16	−0.3	755.3882	45.31	2.18×10^7	−0.419
142	K.DGRTGQPGAVGPAGIR.G	20.04	1507.7855	16	0.1	503.6025	27.13	3.62×10^6	−0.6
143	S.TGEIGPAGPPGPP(+31.99)GLR.G	19.78	1503.7681	16	−0.3	752.8911	39.42	1.52×10^7	−0.537
144	R.GPSGPQGPSGPP(+15.99)GPKGN.S	26.56	1502.7113	17	0.1	752.363	17.77	5.42×10^6	−1.441
145	G.KDGRTGQP(+15.99)GAVGPAGI.R	17.35	1495.7743	16	−0.2	499.5986	24.52	1.74×10^6	−0.562
146	K.STGISVPGPMGPSGPR.G	19.78	1495.7452	16	0.1	748.88	41.79	3.77×10^7	−0.312
147	R.GLP(+15.99)GADGRAGVMGPAGS.R	17.17	1484.7041	17	−1	743.3586	41.88	6.34×10^6	0.053
148	A.RGSDGSVGPVGPAGPIG.S	20.94	1478.7477	17	−0.8	740.3806	41.68		−0.124
149	G.AVGPAGAVGPRGPSGPQ.G	19.51	1473.7688	17	−0.7	737.8912	29.9	5.84×10^6	−0.2
150	G.AVGPAGPRGPAGPSGPAG.K	22.45	1471.7531	18	−0.1	736.8837	26.25	6.23×10^7	−0.239
151	G.LVGEP(+15.99)GPAGSKGESGN.K	17.4	1470.6949	16	1.2	736.3557	17.62	3.58×10^5	−0.713
152	R.GNPGSR(+31.99)GLPGADGRAG.V	16.2	1469.697	16	0.4	735.8561	17.26	5.32×10^5	−0.938
153	M.GPRGFPGSP(+31.99)GNIGPAG.K	15.45	1468.7058	16	0.1	735.3602	34.83	1.96×10^8	−0.531
154	M.GPRGFP(+31.99)GSPGNIGPAG.K	15.37	1468.7058	16	0.2	735.3604	32.68	1.96×10^8	−0.531
155	S.TGISVPGPMGPSGPRG.L	17.66	1465.7347	16	1.1	733.8755	48.09	1.25×10^7	−0.287
156	G.ISGPPGPP(+31.99)GPAGKEGL.R	17.98	1461.7463	16	−0.1	731.8804	36	2.18×10^7	−0.506
157	G.ISGPP(+31.99)GPPGPAGKEGL.R	16.87	1461.7463	16	1.2	731.8813	35.65	2.18×10^7	−0.506
158	G.LP(+15.99)GIDGRPGPIGPAGA.R	24.33	1459.7782	16	−1.1	730.8956	51.62	5.50×10^8	0
159	G.GYDFGFDGDFYR.A	20.34	1457.5887	12	0.8	729.8022	83.97	2.03×10^6	−0.867
160	A.GLP(+15.99)GIDGRPGPIGPAG.A	23.56	1445.7626	16	0.3	723.8888	53.15	2.30×10^8	−0.138
161	R.GFPGLP(+15.99)GPSGEPGKQ.G	15.18	1439.7045	15	−0.2	720.8594	45.75	9.51×10^5	−0.9
162	A.Q(−17.03)FDGKGGGPGPMGLM.G	19.16	1430.6322	15	0.2	716.3235	89.1	9.02×10^6	−0.407
163	A.GAPGP(+15.99)QGPVGPTGKHG.S	17.76	1428.7109	16	−0.2	477.2442	16.72	2.41×10^6	−0.881
164	S.TGISVP(+15.99)GPMGPSGPR.G	21.59	1424.7081	15	−0.9	713.3607	43.4	1.49×10^7	−0.28
165	S.TGISVPGPM(+15.99)GPSGPR.G	16.22	1424.7081	15	0.3	713.3616	38.52	1.38×10^7	−0.28
166	S.GPP(+31.99)GPPGPAGKEGLR.G	18.13	1417.7313	15	0.1	473.5844	19.63	2.94×10^7	−1.087

167	G.AVGPAGPRGPAGPSGPA.G	23.29	1414.7317	17	−0.1	708.373	26.72	7.98×10^6	−0.229
168	M.GPRGFPGSP(+31.99)GNIGPA.G	16.77	1411.6843	15	0.2	706.8495	36.48	5.07×10^7	−0.54
169	M.GPRGFP(+31.99)GSPGNIGPA.G	15.88	1411.6843	15	−0.1	706.8494	34.49	5.07×10^7	−0.54
170	S.TGISVPGPMGPSGPR.G	17.51	1408.7133	15	−0.5	705.3636	42.08	4.49×10^6	−0.28
171	A.GAVGPRGPSGPQ(+.98)GIR.G	22	1405.7426	15	0.1	469.5882	23		−0.64
172	A.GAVGPRGPSGPQGIR.G	19.71	1404.7585	15	−0.7	469.2598	22.08	3.61×10^6	−0.64
173	G.AVGPRGPSGPQGIRG.D	16.94	1404.7585	15	−0.4	469.2599	22.53	3.61×10^6	−0.64
174	S.GERGPP(+15.99)GPMGPPGLA.G	20.8	1404.6819	15	−0.5	703.3478	40.37	9.00×10^6	−0.7
175	T.GEIGPAGPPGPP(+31.99)GLR.G	17.38	1402.7205	15	0.4	702.3678	39.29	4.76×10^7	−0.527
176	A.VGPAGPRGPAGPSGPAG.K	20.93	1400.7159	17	−0.1	701.3652	25.84	4.20×10^6	−0.359
177	G.YDFGFDGDFYR.A	21.23	1400.5673	11	−0.1	701.2908	83.82	4.53×10^6	−0.909
178	G.LP(+15.99)GIDGRPGPIGPAG.A	24.36	1388.7411	15	0.3	695.3781	47.55	1.37×10^8	−0.12
179	V.GLP(+15.99)GIDGRPGPIGPA.G	22.29	1388.7411	15	−0.3	695.3776	54	3.04×10^8	−0.12
180	R.GPSGPQGPSGPP(+15.99)GPKG.N	24.16	1388.6683	16	0	695.3414	18.63	1.13×10^6	−1.312
181	P.GFP(+15.99)GARGPSGPQGPS.G	18.55	1383.6531	15	−0.2	692.8337	26.05	4.18×10^6	−0.893
182	A.GPAGPRGEVGLP(+15.99)GLS.G	19.61	1378.7205	15	0.9	690.3681	47.62	1.13×10^7	−0.133
183	E.TGPAGPAGPIGPVGAR.G	21.9	1373.7415	16	−0.1	687.8779	36.93	2.46×10^7	0.031
184	A.GAPGP(+15.99)QGPVGPTGKH.G	18.23	1371.6895	15	−0.3	458.237	16.98	8.29×10^5	−0.913
185	G.I(+57.02)SVPGPMGPSGPRG.L	17.79	1364.687	14	−0.1	683.3507	45.43	3.55×10^7	−0.25
186	T.GISVPGPMGPSGPRG.L	16.81	1364.687	15	−0.2	683.3506	45.85	3.55×10^7	−0.26
187	T.G(+57.02)ISVPGPMGPSGPR.G	15.5	1364.687	14	0.6	683.3512	47.23	2.63×10^6	−0.25
188	G.SRGLP(+15.99)GADGRAGVM.G	22.91	1358.6725	14	−0.7	453.8978	27.35	7.41×10^6	−0.214
189	G.AVGPRGPSGPQ(+.98)GIR.G	23.59	1348.7211	14	−0.3	450.5808	23.16	7.60×10^5	−0.657
190	G.AVGPRGPSGPQGIR.G	20.94	1347.7371	14	−0.8	450.2526	21.33	8.22×10^6	−0.657
191	R.GVQGPP(+15.99)GPAGPRGAN.G	19.72	1346.6691	15	0.1	674.3419	19.38	1.61×10^6	−0.807
192	A.GVMGPAGSRGATGPAG.V	20.27	1341.6459	16	0.7	671.8307	23.94	1.88×10^6	−0.006
193	R.GGP(+15.99)GSRGFPGSDGVA.G	16.63	1332.6058	15	0.3	667.3104	28.32	2.48×10^7	−0.427
194	G.LP(+15.99)GIDGRPGPIGPA.G	23.12	1331.7197	14	0.5	666.8675	51.06	3.15×10^8	−0.1
195	Q.GIAGQRGVVGLP(+15.99)GQ.R	21.29	1323.7258	14	0.3	662.8704	38.51	1.51×10^7	0.243
196	R.GIP(+15.99)GPVGAAGATGARG.L	19.66	1323.6895	16	0.3	662.8522	29.04	2.55×10^7	0.319
197	T.GISVP(+15.99)GPMGPSGPR.G	20.17	1323.6605	14	0.3	662.8378	40.58	3.35×10^7	−0.25
198	T.GISVPGPM(+15.99)GPSGPR.G	16.25	1323.6605	14	−0.1	662.8375	36.4	2.27×10^7	−0.25
199	H.GDQGAP(+15.99)GAVGPAGPR.G	20.67	1321.6375	15	0.2	661.8261	18.32	1.50×10^6	−0.58
200	T.GPAGPP(+31.99)GFPGAVGAK.G	17.14	1310.6619	15	0.2	656.3383	33.75	5.65×10^6	0.007
201	K.GETGPSGPAGPTGAR.G	17.49	1310.6215	15	−0.1	656.3179	17.96	3.44×10^5	−0.893
202	G.I(+57.02)SVPGPMGPSGPR.G	19.13	1307.6655	13	0.6	654.8405	45.94	2.27×10^8	−0.238
203	G.ISVPGPMGPSGPRG.L	17.73	1307.6655	14	0.7	654.8405	43.27	5.27×10^7	−0.25
204	V.GPAGPRGPAGPSGPAG.K	20.19	1301.6476	16	−0.8	651.8306	20.36		−0.644
205	A.P(+15.99)GPQGPVGPTGKHG.S	17.23	1300.6523	14	−0.2	434.558	16.86	3.06×10^5	−1.107
206	G.V(+57.02)MGPAGSRGATGPA.G	18.2	1284.6244	14	0	643.3195	24.23		0.05
207	G.VMGPAGSRGATGPAG.V	16.82	1284.6244	15	0.7	643.3199	21.31	2.21×10^6	0.02
208	G.A(+57.02)RGSDGSVGPVGPA.G	17.93	1282.6266	14	−0.3	642.3204	22.2	3.26×10^6	−0.171

209	A.GARGSDGSVGPVGPA.G	18.46	1282.6265	15	0.2	642.3206	23.02	3.26×10^{6}	−0.187
210	T.GPAGPAGPIGPVGAR.G	20.08	1272.6938	15	0	637.3542	34.76	2.18×10^{7}	0.08
211	G.FPGLP(+31.99)GPSGEPGK.Q	15.64	1270.6193	13	−0.2	636.3168	40.61	8.60×10^{6}	−0.738
212	G.IAGQRGVVGLP(+15.99)GQ.R	20.36	1266.7043	13	−0.3	634.3593	33.38	2.78×10^{7}	0.292
213	R.GIP(+15.99)GPVGAAGATGAR.G	17.47	1266.668	15	0	634.3412	28.9	4.42×10^{6}	0.367
214	G.ISVP(+15.99)GPMGPSGPR.G	21.61	1266.639	13	−0.6	634.3264	38.05	7.78×10^{6}	−0.238
215	G.ISVPGPM(+15.99)GPSGPR.G	15.97	1266.639	13	−0.5	634.3265	34.48	3.52×10^{7}	−0.238
216	G.DQGAP(+15.99)GAVGPAGPR.G	17.59	1264.616	14	0.4	633.3155	19.25	8.49×10^{5}	−0.593
217	S.GPP(+31.99)GPPGPAGKEGL.R	16.11	1261.6302	14	0.2	631.8225	26.16	7.44×10^{6}	−0.843
218	P.GFLGLP(+15.99)GSRGER.G	15.56	1260.6575	12	−0.3	421.2263	35.18	1.75×10^{6}	−0.508
219	G.ISVPGPMGPSGPR.G	19.71	1250.644	13	−0.4	417.8885	43.78	5.85×10^{6}	−0.238
220	P.GIDGRP(+15.99)GPIGPAGA.R	19.15	1249.6415	14	−0.1	625.8279	30.9	1.88×10^{6}	−0.157
221	G.A(+57.02)VGPAGAVGPRGPS.G	18.9	1248.6575	14	−0.8	625.3355	27.2	3.39×10^{6}	0.15
222	R.GVVGLP(+15.99)GQRGER.G	19.48	1239.6683	12	−0.4	620.8412	19.99	2.06×10^{6}	−0.583
223	Y.DFGFDGDFYR.A	15.86	1237.5039	10	0.4	619.7595	76.55	1.59×10^{7}	−0.87
224	K.GIRGHN(+.98)GLDGLK.G	21.16	1236.6575	12	−0.4	413.2263	20.56	2.19×10^{6}	−0.675
225	E.RGPPGPM(+31.99)GPPGLA.G	16.54	1234.6128	13	−0.6	618.3133	29.81	4.23×10^{6}	−0.508
226	G.I(+57.02)DGRPGPIGPAGA.R	19.07	1233.6465	13	0.3	617.8307	36.48	5.06×10^{6}	−0.138
227	G.VMGPAGSRGATGPA.G	16.08	1227.603	14	−1.8	614.8077	21	6.94×10^{5}	0.05
228	E.RGPP(+15.99)GPMGPPGLA.G	20.53	1218.6179	13	0	610.3162	37.65	4.38×10^{7}	−0.508
229	R.GQAGVMGFP(+15.99)GPKG.A	19.26	1217.5863	13	0.6	609.8008	43.49	1.29×10^{7}	−0.146
230	G.PAGPAGPIGPVGAR.G	20.5	1215.6724	14	0.6	608.8438	33.28	2.35×10^{6}	0.114
231	G.FLGLP(+15.99)GSRGER.G	16.42	1203.636	11	0.2	602.8254	33.39	4.72×10^{6}	−0.518
232	R.TGQP(+15.99)GAVGPAGIR.G	20.23	1195.6309	13	0.4	598.8229	23.06	6.91×10^{5}	−0.092
233	E.FGFDGDFYRA.D	15.97	1193.5142	10	0.1	597.7644	75.78	2.26×10^{7}	−0.34
234	G.AVGPRGPSGPQGI.R	18.46	1191.636	13	−0.4	596.825	30.76	9.34×10^{5}	−0.362
235	G.AVGPAGPRGPAGPS.G	19.27	1189.6204	14	−0.3	595.8173	21.68	1.21×10^{6}	−0.264
236	G.VVGLP(+15.99)GQRGER.G	18.17	1182.6469	11	0.2	592.3308	18.12	2.12×10^{6}	−0.6
237	G.IRGHN(+.98)GLDGLK.G	22.45	1179.636	11	−0.2	394.2192	18.73	4.44×10^{6}	−0.7
238	R.TGQPGAVGPAGIR.G	17.45	1179.636	13	0.2	590.8254	26.21	8.29×10^{5}	−0.092
239	G.IRGHN(+.98)GLDGLK.G	22.45	1179.636	11	−0.2	394.2192	18.73	4.44×10^{6}	−0.7
240	P.GIDGRP(+15.99)GPIGPAG.A	15.18	1178.6044	13	0.1	590.3095	27.06	9.47×10^{5}	−0.308
241	G.LP(+15.99)GIDGRPGPIG.P	17.48	1163.6298	12	0.1	582.8222	48.07	4.57×10^{6}	−0.133
242	R.GVQGPP(+15.99)GPAGPRG.A	18.5	1161.589	13	0.4	581.802	18.82	1.59×10^{6}	−0.8
243	G.QAGVMGFP(+15.99)GPKG.A	19.62	1160.5648	12	0.2	581.2898	44.91		−0.125
244	A.GPRGEVGLP(+15.99)GLS.G	17.07	1153.6091	12	0.8	577.8123	42.11	3.56×10^{7}	−0.15
245	G.A(+57.02)VGPRGPSGPQG.I	19.41	1135.5734	12	0.1	568.794	17.32	1.03×10^{7}	−0.767
246	A.GAVGPRGPSGPQG.I	19.07	1135.5734	13	0.1	568.794	18.46	1.03×10^{7}	−0.738
247	D.FGFDGDFYR.A	15.89	1122.4771	9	0.4	562.246	67.98	1.05×10^{7}	−0.578
248	R.GLP(+15.99)GADGRAGVM.G	16.66	1115.5393	12	0.1	558.777	34.78	4.24×10^{7}	0.192
249	P.GIDGRPGPIGPA.G	15.01	1105.5879	12	1.4	553.802	34.27	1.20×10^{6}	−0.3
250	R.GVQGPP(+15.99)GPAGPR.G	18.55	1104.5675	12	0.2	553.2911	18.86	9.21×10^{5}	−0.833

251	G.NIGFP(+15.99)GPKGPT.G	16.82	1099.5662	11	0.9	550.7908	35.91	6.63×10^6	−0.618
252	I.GQP(+15.99)GAVGPAGIR.G	19.44	1094.5833	12	0.1	548.299	22.73	2.36×10^6	−0.042
253	R.GPPGP(+31.99)MGPPGLA.G	16.91	1078.5117	12	0.3	540.2633	38.38	1.89×10^7	−0.175
254	R.GPP(+15.99)GPMGPPGLA.G	19.98	1062.5167	12	0.3	532.2658	48.83	1.40×10^8	−0.175
255	G.LMGPRGPP(+15.99)GAS.G	18.84	1054.5229	11	0.2	528.2689	22.6	7.21×10^5	−0.345
256	S.VPGPMGPSGPR.G	16.37	1050.5281	11	0.1	526.2714	25.34	8.56×10^6	−0.618
257	G.LTGPIGPP(+15.99)GPAG.A	16.75	1048.5553	12	−0.1	525.2849	40.72	2.49×10^7	0.117
258	R.GLTGPIGPP(+15.99)GPA.G	16.31	1048.5553	12	0.7	525.2853	48.25	4.62×10^7	0.117
259	A.GPAGPIGPVGAR.G	15.29	1047.5825	12	0.2	524.7986	27.82	1.78×10^7	0.117
260	N.IGFP(+15.99)GPKGPTG.D	17.57	1042.5447	11	−0.3	522.2795	35.5	8.40×10^6	−0.336
261	G.QRGVVGLP(+15.99)GQ.R	17.88	1025.5618	10	0.1	513.7882	26.19	9.65×10^5	−0.21
262	G.AVGPRGPSGPQ.G	16.59	1021.5305	11	−0.3	511.7724	16.92	2.85×10^6	−0.8
263	G.A(+57.02)VGPAGAVGPR.G	15.52	1007.5512	11	0.5	504.7831	22.78	6.94×10^5	0.445
264	G.LP(+15.99)GIDGRPGP.I	16.09	993.5243	10	−0.1	497.7694	37.02	6.23×10^6	−0.57
265	G.LTGPIGPP(+15.99)GPA.G	18.09	991.5338	11	0.7	496.7745	41.07	2.04×10^8	0.164
266	N.IGFP(+15.99)GPKGPT.G	17.85	985.5233	10	−0.2	493.7688	34.49	1.88×10^7	−0.33
267	A.GVMGFP(+15.99)GPKG.A	17.29	961.4691	10	−0.2	481.7418	38.9	3.95×10^7	0.02
268	R.GVVGLP(+15.99)GQRG.E	15.19	954.5247	10	−0.2	478.2695	23.86	3.10×10^6	0.1
269	G.VMGFP(+15.99)GPKG.A	17.07	904.4476	9	0.2	453.2312	35.24	1.55×10^7	0.067
270	Q.RGVVGLP(+15.99)GQ.R	16.34	897.5032	9	−0.4	449.7587	25.94		0.156
271	L.TGPIGPP(+15.99)GPA.G	16.83	878.4498	10	0	440.2321	27.77	1.61×10^7	−0.2
272	R.GPP(+15.99)GPMGPPG.L	15.63	878.3956	10	0.5	440.2053	25.51	2.01×10^6	−0.77

* -lg10P, The peptide score (-10lgP) is calculated for every peptide-spectrum match (PSM) reported by PEAKS DB, PEAKS PTM, and SPIDER. The score is derived from the *p*-value that indicates the statistical significance of the peptide-spectrum match. A peptide may be matched to many spectra, resulting in multiple PSMs. In that case, the peptide's score is calculated as the maximum of all the PSMs. *The grand average of hydropathicity (GRAVY) index value was used to evaluate the hydrophilic and hydrophobic character of the identified peptides
